# A Systematic Review of Melatonin in Plants: An Example of Evolution of Literature

**DOI:** 10.3389/fpls.2021.683047

**Published:** 2021-06-18

**Authors:** Susan J. Murch, Lauren A. E. Erland

**Affiliations:** Department of Chemistry, University of British Columbia, Kelowna, BC, Canada

**Keywords:** melatonin, indoleamine, plant signaling, morphogenesis, development, stress

## Abstract

Melatonin (N-acetyl-5-methoxy-tryptamine) is a mammalian neurohormone, antioxidant and signaling molecule that was first discovered in plants in 1995. The first studies investigated plant melatonin from a human perspective quantifying melatonin in foods and medicinal plants and questioning whether its presence could explain the activity of some plants as medicines. Starting with these first handful of studies in the late 1990s, plant melatonin research has blossomed into a vibrant and active area of investigation and melatonin has been found to play critical roles in mediating plant responses and development at every stage of the plant life cycle from pollen and embryo development through seed germination, vegetative growth and stress response. Here we have utilized a systematic approach in accordance with the preferred reporting items for systematic reviews and meta-analyses (PRISMA) protocols to reduce bias in our assessment of the literature and provide an overview of the current state of melatonin research in plants, covering 1995–2021. This review provides an overview of the biosynthesis and metabolism of melatonin as well as identifying key themes including: abiotic stress responses, root development, light responses, interkingdom communication, phytohormone and plant signaling. Additionally, potential biases in the literature are investigated and a birefringence in the literature between researchers from plant and medical based which has helped to shape the current state of melatonin research. Several exciting new opportunities for future areas of melatonin research are also identified including investigation of non-crop and non-medicinal species as well as characterization of melatonin signaling networks in plants.

## Introduction

**FIGURE 1:**
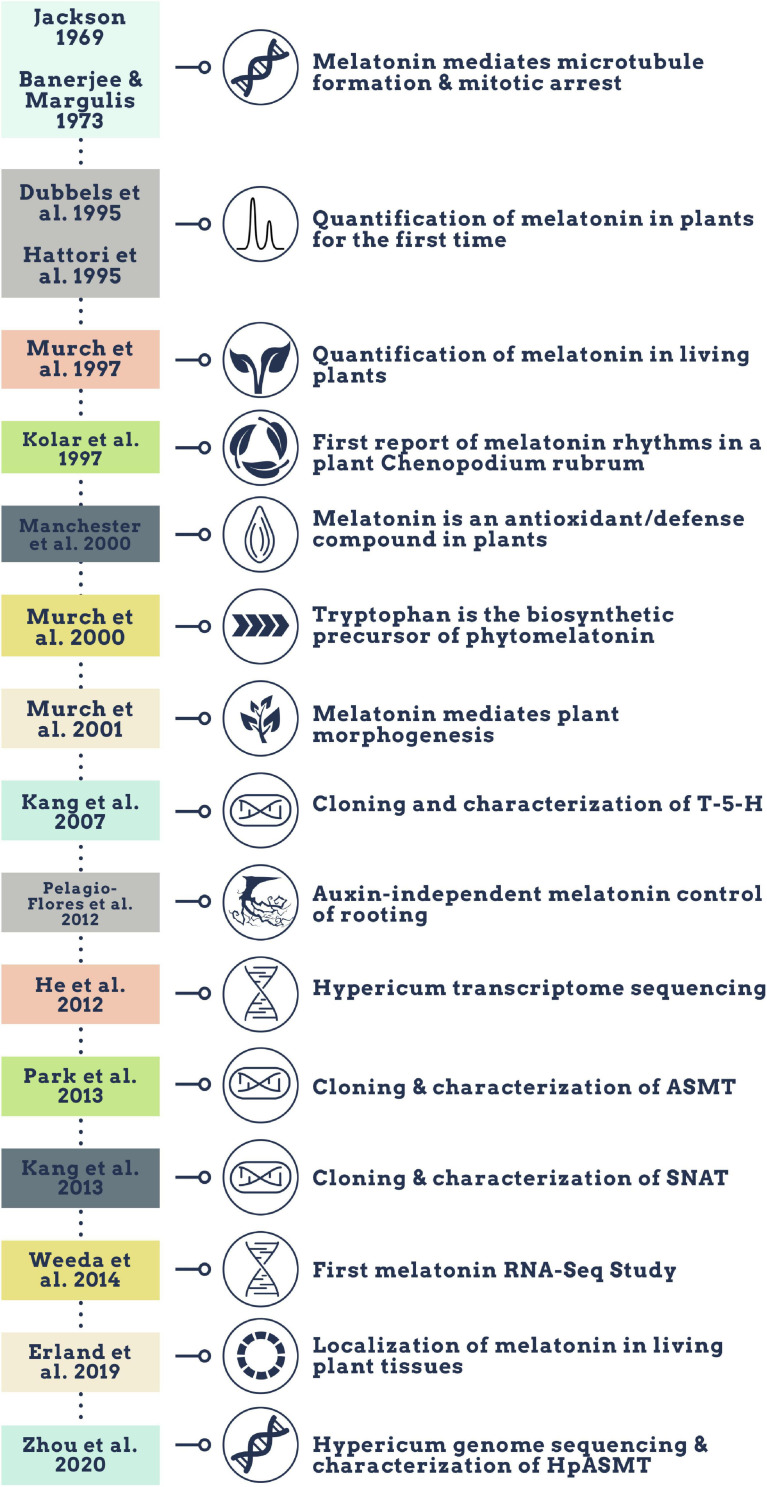
Timeline of qualitative major melatonin research developments.

**FIGURE 2:**
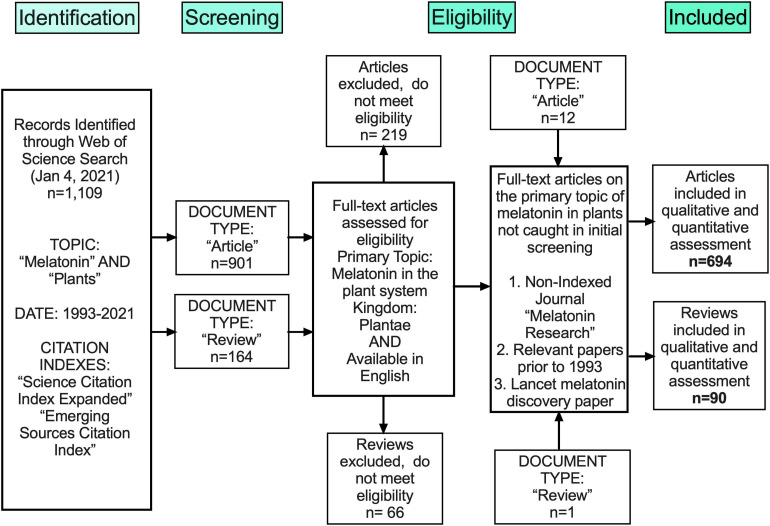
Flow chart outlining methods of unbiased collection of literature following the PRISMA protocol.

**FIGURE 3:**
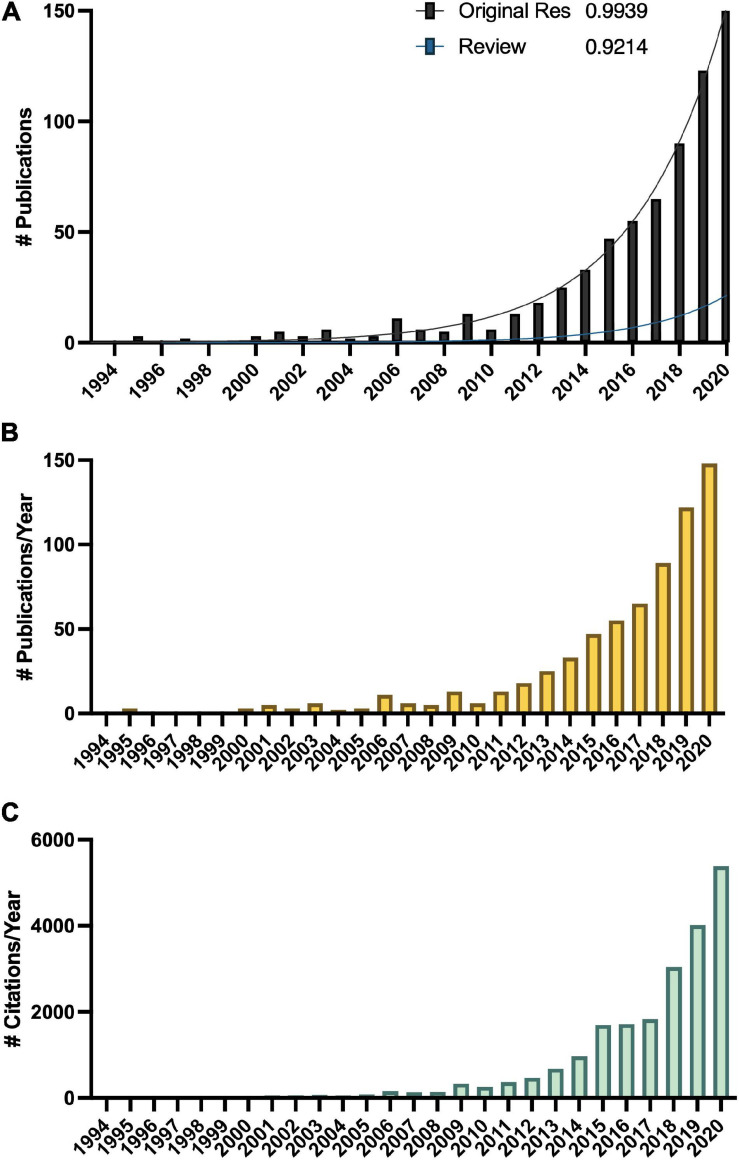
Growth in the melatonin literature **(A)** total number of publications, with *R*^2^ indicating best fit to an exponential curve, **(B)** total number published per year, and **(C)** total number of citations of articles captured in this analysis per year according to Web of Science.

Melatonin (N-acetyl-5-methoxy-tryptamine) is a mammalian neurohormone, antioxidant and signaling molecule that was first discovered in plants in 1995 (Dubbels et al., 1995; Hattori et al., 1995). The first studies investigated plant melatonin from a human perspective quantifying melatonin in foods (Dubbels et al., 1995; Hattori et al., 1995) and medicinal plants (Manchester et al., 2000), and questioning whether its presence could explain the activity of some plants as medicines (Murch et al., 1997; Chen et al., 2003). Starting with these first handful of studies in the late 1990s, plant melatonin research has blossomed into a vibrant and active area of investigation (Figure 1) with the first 2 papers cited > 530 times each. These 2 papers provide a set starting point for an evaluation of how the scientific literature evolved in melatonin research (Figure 1). The initial goal of this systematic review was to understand the important roles of melatonin in plant physiology, growth, and metabolism. Application of network analysis approaches identified that divergent research groups have led investigations in divergent directions that influenced the relative weight of research outcomes. We have used a systematic approach to this review following the Preferred Reporting Items for Systematic Reviews and Meta-Analyses (PRISMA) protocols to reduce bias in our assessment of the literature (Figure 2) and a full bibliography is included in the Supplementary Materials (Supplementary Tables 1, 2). Our goal was to assess how the literature in the field has developed over the last 25 years. Some of the potential biases in our data include: (a) only manuscripts published in English were included, (b) our search was limited to primarily literature from peer-reviewed and indexed publications and (c) reports and data from governments, regulatory agencies, public or private corporations or proprietary processes were not included. Our assessment of the literature covers publications that appeared between 1995 – 2021. We found 692 manuscripts containing original research data and results describing distribution, metabolism, and mechanisms of melatonin in plants. A further 93 manuscripts are reviews of aspects of the literature from a viewpoint. Interestingly, the last 5 years have been particularly active in plant melatonin research with an exponential growth in publications (*R*^2^ = 0.9939, Figure 3). Over time, the number of review papers on the topic of plant melatonin has kept pace with primary original research (*R*^2^ = 0.914, Figure 3). In total, 785 scientific publications were assessed in this systematic review. The major developments are shown in a timeline of keystone papers (Figure 1).

## Birefringence in the Literature

**FIGURE 4:**
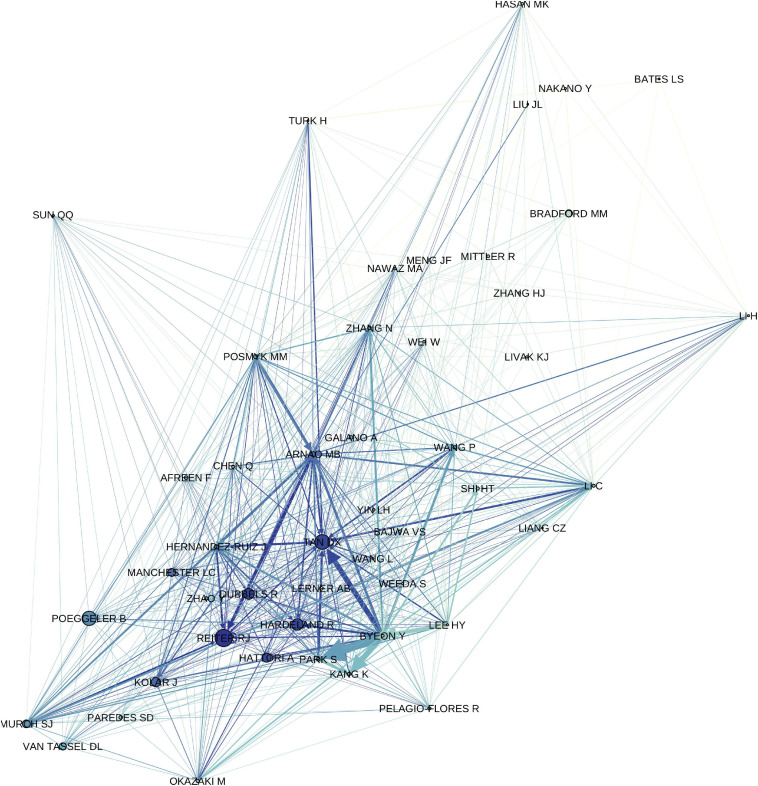
Author correlation network showing relationship between first and last authors of original research articles included in the study and first authors of citing articles, filtered to an in-degree cutoff of >100. Increasing edge thickness indicates a stronger relationship (higher edge weight), edges are colored to match target node, increasing node size indicates higher page rank (network importance), nodes are colored based on in-degree, i.e., number of authors citing with authors with a greater number of citations being darker.

Our research finds that over time, the plant melatonin community diverged into dichotomous fields and clusters of authors (Figure 4). In general, researchers with backgrounds in medicine and human health where melatonin is the master regulator of circadian rhythms look to mammalian systems for inspiration and initially focused on understanding the implications of dietary sources of melatonin on humans (Dubbels et al., 1995; Hattori et al., 1995; Reiter et al., 2005). This has been a productive area of research and has made significant advancements in understanding the health outcomes of humans and animals consuming plants containing high amounts of melatonin as foods and/or medicines. The role of melatonin as a neurohormone in humans led to significant initial interest in melatonin as a bioactive or in synergy with other bioactives in medicinal plants, herbal products and dietary supplements including sleep aids [e.g., valerian (*Valeriana officinalis* L.), passionflower (*Passiflora incarnata* L.), chamomile (*Matricaria chamomilla* L.), ashwagandha (*Withania somnifera* (L.) Dunal)], psychoactive plants (e.g., *Datura metel* L., *Cannabis sativa* L., *Papaver somniferum L*.) and treatment of neurological conditions, such as feverfew (*Tanacetum parthenium* L.) in the treatment of migraines (Vogler et al., 1998; Murch et al., 2009; Allegrone et al., 2019). Plant-based melatonin as a vegan supplement is growing in popularity, particularly in light of surging demands for melatonin for the treatment of coronavirus disease-19 (COVID19; Arnao and Hernández-Ruiz, 2018b; Arnao and Hernández-Ruiz, 2019b; Pérez-Llamas et al., 2020). A second stream of investigation has been led by researchers with expertise in the plant sciences who were inspired by the structural similarity between melatonin and the plant growth regulator indole-3-acetic acid (IAA; auxin). Research along these lines has led to significant advancements in understanding the roles of melatonin in root growth, seed germination and control of plant morphology (Pelagio-Flores et al., 2012; Zhang et al., 2014; Erland et al., 2018). Another avenue of investigation seems to have evolved with an emphasis on the ecological implications of plant melatonin and the potential roles of melatonin in adaptation, evolution and mitigating environmental stresses (Tan et al., 2014; Arnao and Hernández-Ruiz, 2019c). In this review, we have attempted to bring together divergent approaches and viewpoints to understand the synergy between approaches and the potential for discovery in the intersections between disciplines (Figure 4).

## Biosynthesis of Plant Melatonin via Multiple Diverse and Redundant Pathways

**FIGURE 5:**
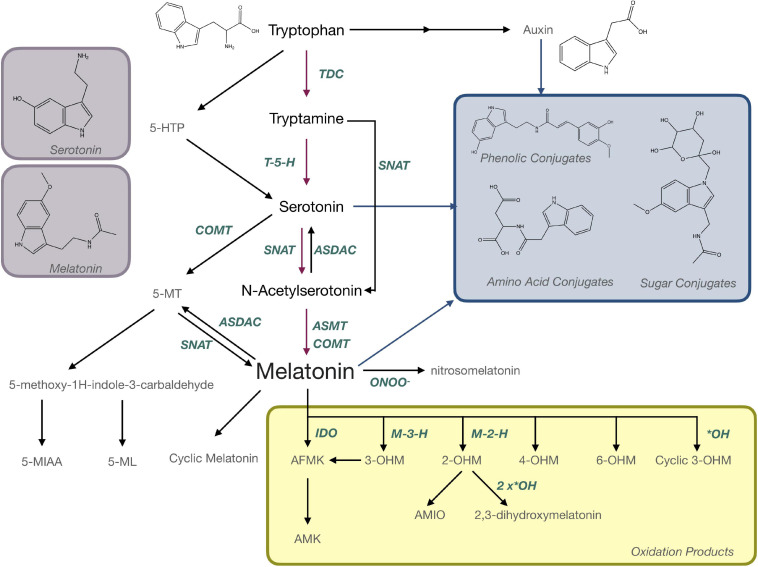
Biosynthesis and metabolism of melatonin in plants. AFMK, N-acetyl-N-formyl-5-methoxykynuramine; AMIO, 3-acetamidoethyl-5-methoxyindolin-2-one; AMK, N-acetyl-5-methoxykynuramine; ASDAC, N-acetylserotonin deacetylase; ASMT, N-acetylserotonin-O-methyltransferase; COMT, caffeic acid-O-methyltransferase; 5-HTP, 5-hydroxytryptophan; IDO, indoleamine 2,3-dioxygenase; M-3-H, melatonin 3-hydroxylase; M-2-H, melatonin 2-hydroxylase; 5-MIAA, 5-methoxyindole-3-acetaldehyde; 5-ML, 5-methyoxytryptophol; 5-MT; OHM, hydroxymelatonin; SNAT, serotonin N-acetyltransferase; TDC, tryptophan decarboxylase.

Evidence suggesting that melatonin in plants is produced from the aromatic amino acid tryptophan was first reported 20 years ago (Figure 1) where radiolabeling studies using ^14^C-tryptophan demonstrated rapid conversion to melatonin in under an hour in *in vitro* grown plantlets (Murch et al., 2000). Over the last 20 years, molecular approaches have elucidated a full pathway for biosynthesis with several alternate mechanisms (Figure 5). In the primary pathway tryptophan is first converted to tryptamine through a decarboxylation reaction catalyzed by tryptophan decarboxylase (TDC; Zhou et al., 2020). Interestingly, this mechanism may not be conserved through evolution in all plant species since TDC1 is absent from some ecotypes of *Arabidopsis* and indeed different ecotypes of *Arabidopsis* have been found to respond differentially to melatonin exposure (Zia et al., 2019). Tryptamine is then hydroxylated to serotonin (5HT; 5-hydroxytryptamine) by tryptamine-5-hydroxylase (Kang et al., 2007). While TDC is highly regulated in most plant species, the conversion of tryptamine to 5HT appears to occur rapidly and with little feedback or regulation, aside from competition for tryptamine, which also serves as a precursor for many secondary metabolic pathways (Kang et al., 2007; Erland et al., 2018). In mammals, 5-hydroxytryptophan (5-HTP) is an intermediate between tryptamine and 5HT, but this part of the mechanism is not as well understood in plants. Quantification of 5-HTP in plants suggests it may be involved in 5HT biosynthesis or catabolism but an enzyme catalyzing these reactions has not been identified at the time of writing this review (Reynolds et al., 1983). Biosynthesis of melatonin from 5HT occurs through two main intermediates; (1) N-acetylserotonin (NAS), in a reaction catalyzed by serotonin-N-acetyltransferase (SNAT; Kang et al., 2013) following the same pathway as in animals, or (2) 5-methoxytryptamine (5-MT), which is catalyzed by a caffeic acid-O-methyltransferase (COMT; Lee et al., 2014b; Figure 5). SNAT can use tryptamine as a substrate for NAS production, skipping the need for production of 5HT, and can also catalyze the acetylation of 5-MT to form melatonin (Byeon et al., 2013; Figure 5). Recently, a novel deacetylase enzyme, NAS deacetylase (ASDAC), has been characterized that catalyzes the reverse reactions converting NAS to 5HT, or melatonin to 5-MT allowing for the possibility of interconversion between 5HT and melatonin (Lee et al., 2018). The final step in the primary pathway is methylation of NAS to melatonin catalyzed by the enzyme NAS methyl transferase (ASMT; Park et al., 2013a), or COMT (Byeon et al., 2014; Figure 5). Melatonin biosynthesis has been shown to occur in both the chloroplast (Zheng et al., 2017) and the mitochondria (Wang et al., 2017) with some possible cytosolic involvement. To date, melatonin transport proteins have not been characterized in plants and this is an area in which further research efforts are warranted. The significant diversity that exists in the melatonin biosynthetic pathway leaves open many areas of future research. More investigations are needed to understand the significance of alternate pathways, evolutionary conservation, divergence or convergence and diversity between families and/or species. The multiple biosynthetic mechanisms create significant complications for studies to create enzyme specific mutants or to manipulate specific genes to determine function as dormant or redundant pathways are activated to recover functions.

## Melatonin Is a Precursor for Bioactive Metabolites

Melatonin is metabolized to produce a number of important bioactive molecules in plants that can generally be grouped as; (a) products of oxidation reactions, (b) products of catabolism and (c) conjugates and derivatives (Figure 5). Nitrosomelatonin, N-acetyl-N-formyl-5-methoxykynuramine (AFMK) and N-acetyl-5-methoxykynuramine (AMK) are products of oxidation reactions as are several of the 5-MT derivatives (Figure 5). AFMK and AMK were the first melatonin metabolites described in plants (Tan et al., 2007a) and function as antioxidants with the capacity to quench reactive oxygen (ROS) and reactive nitrogen species (RNS; Tan et al., 2007b; Schaefer and Hardeland, 2009). It has been proposed that AFMK may be produced by both enzymatic and non-enzymatic reactions, particularly conversion by indole-2,3-dioxygenase (IDO; Okazaki et al., 2010). This hypothesis is still somewhat controversial as other authors argue that tryptophan is generally the substrate for IDO and can react with RNS or ROS (Hardeland, 2014). The levels of AFMK have also been found to vary in coordination with melatonin (Tan et al., 2007a) making it unlikely that melatonin is the primary precursor. Evidence of the function of 5-MT metabolites, 5-methyoxytryptophol (5-ML) and 5-methoxyindole-3-acetaldehyde (5-MIAA) have been investigated to an even lesser extent in plants, though their role as catabolism products of melatonin in dinoflagellates is fairly well described (Hardeland, 2014). Nitrosomelatonin as well as melatonin oxidation products, play important roles as antioxidants in plant, while conjugates have been hypothesized to play roles as sequestration or storage forms. This is the case for phenolic-conjugates of the melatonin precursor, 5HT, which play roles in defense (Ishihara et al., 2008), though the presence and function of melatonin-phenolic compounds is as yet not experimentally confirmed (Erland et al., 2020b). Though the presence of melatonin metabolites is well accepted, their presence and functions in plants have generally not been widely investigated. “Hydroxymelatonin” and “isomer” are mentioned in 14 titles/abstracts each, with the most mentioned, 2-hydroxymelatonin (2-OHM) still only being mentioned in 12 abstracts/titles. Several articles have proposed that some metabolites, particularly 2-OHM and 3-hydroxymelatonin (3-OHM; Byeon et al., 2015b) which are produced by melatonin 2-hydroxylase (M2H; Byeon and Back, 2015) and melatonin 3-hydroxylase (M3H; Lee et al., 2016), respectively, may actually be the predominant forms of melatonin in plants. For example, 2-OHM is reported to confer tolerance to abiotic stresses, such as cold (Lee and Back, 2016b; Back, 2020), drought (Lee and Back, 2016b; Back, 2020), salt (Choi and Back, 2019b), and heavy metal stress (Byeon et al., 2015a; Shah et al., 2019), through both direct antioxidant function and upregulation of antioxidant enzymes (Shah et al., 2019, 2020). 2-OHM may also activate mitogen-activated protein kinase (MAPK) signaling cascades (Lee and Back, 2016a) and modulate gene expression including stress related transcription factors and transport proteins (Lee and Back, 2016b). With so many potential roles and interactions, further investigation of the functions of these metabolites and isomers is warranted. These future investigations should include not just quantification of these compounds but also aim to understand the roles of these metabolites in plants metabolism.

## Melatonin Is an Antioxidant

The story of melatonin as an antioxidant is often repeated and comparatively well understood. The antioxidant potential of melatonin is postulated as a mechanism for stress responses, as well as protection of photosynthetic apparatus and reproductive development/germ tissues. At least 51 of the publications we assessed mention melatonin and ROS specifically. Readers who are interested in further detail are referred to a proliferation of reviews on this topic published in the last 2 years (*n* = 11 published in 2020, 2021; Supplementary Table 2). Interest in this area of research is driven by the hypothesis that the strong antioxidant potential of plant melatonin conferred an evolutionary advantage to the first living organisms on Earth during the great oxygen epoch (Tan et al., 2014; Tan and Reiter, 2020). Mechanistically, melatonin quenches up to 10 units of ROS per molecule and requires no recycling mechanism (Rodriguez-Naranjo et al., 2012). Isomers and metabolites of melatonin including AFMK, AMK and 3-OHM have all been shown to be capable of scavenging free radicals through hydrogen atom transfer (HAT), single electron transfer (SET) and radical adduct formation (RAF) mechanisms and to form a unique free radical scavenging cascade which does not require enzymatic activity (Reina and Martínez, 2018). Melatonin can react with both ROS and RNS to form oxidized or nitrogenated metabolites. For example, reaction of melatonin with the hydroxyl radical yields 2,3-dihydroxymelatonin and cyclic 3-OHM (Back, 2020), while reaction with oxoperoxonitrate (ONOO^–^) yields 1-nitrosomelatonin (Mukherjee, 2019). The capacity to neutralize ROS also plays an important role in melatonin’s control of ROS and reactive nitrogen species (RNS) signaling networks within plants, in particular hydrogen peroxide (H_2_O_2_) and nitric oxide (NO) signaling networks which allow for rapid signaling in response to stimulus (Lee and Back, 2017).

## Precise, Accurate and Sensitive Quantification of Melatonin Reveals Isomers

**FIGURE 6:**
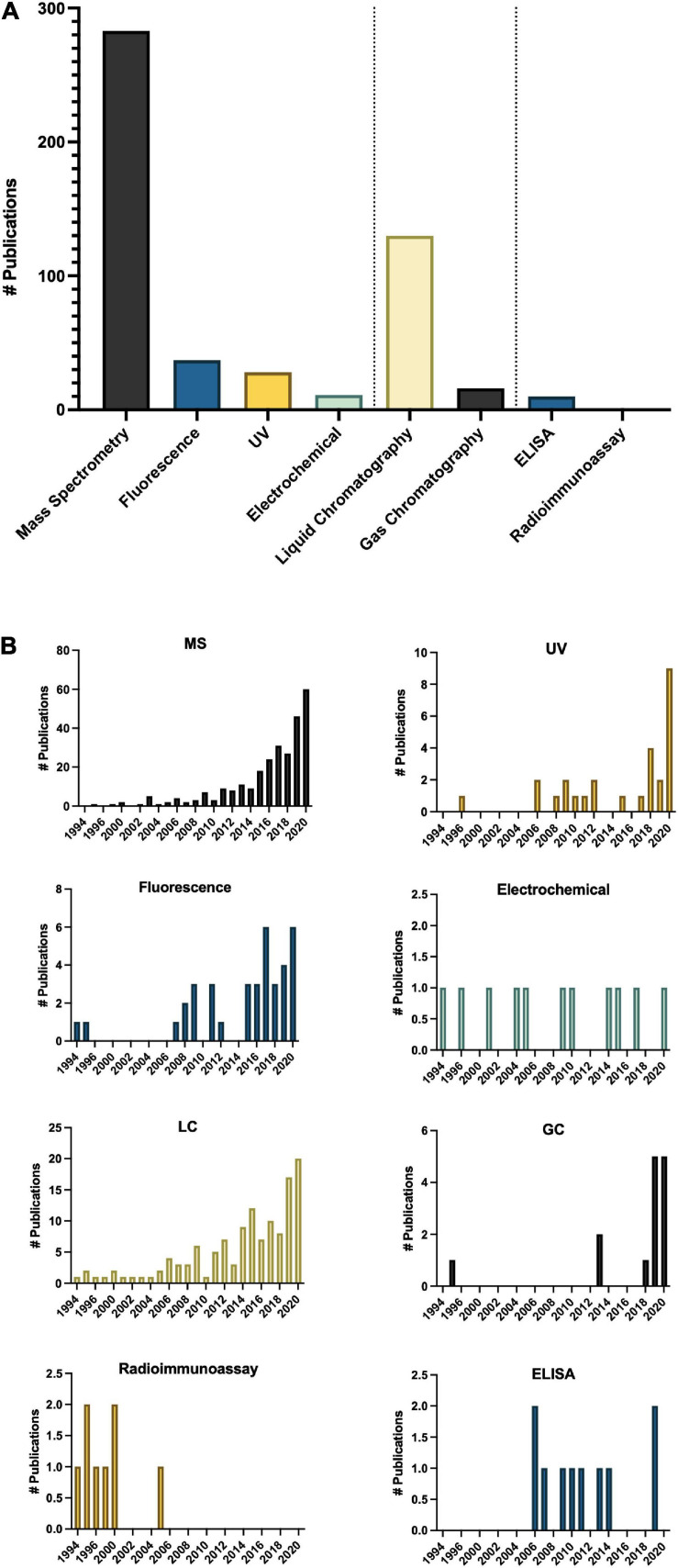
Methods of analysis of melatonin in plants **(A)** total number of publications by technique over the time period queried and **(B)** Number of publications by technique by year. ELISA, enzyme-linked immunosorbent assay; GC, gas chromatography; LC, liquid chromatography; MS, mass spectrometry; UV, ultra violet detection.

Methodologies for detecting and quantifying endogenous melatonin have seen a significant shift since 1995 (Dubbels et al., 1995; Hattori et al., 1995; Figure 6). The first studies quantifying melatonin in plants used antibody-based techniques, namely radioimmunoassay (RIA; Dubbels et al., 1995; Hattori et al., 1995), however, mass spectrometry (MS)-based techniques including quadrupole, time of flight and tandem systems now make up the majority of methods (Figure 6). Development of new technologies are reflected in the trends of preferred analytical method for example a shift from RIA to enzyme-linked immunosorbent assays (ELISA; Figure 6). Overwhelmingly, the preferred approach for quantification of melatonin has become liquid chromatography (LC)-MS based approaches (Figure 6). In addition to the higher sensitivity and specificity of LC-MS based approaches, this increase is also likely due to the increased ease of use of these platforms, a greater number of published protocols and decreasing cost of entry. LC-MS approaches generally also have improved ease of use and higher throughput as compared to electrochemical detection methods which often require long equilibration times and or gas-chromatography (GC) based methods which require derivatization prior to analysis. Another advantage to LC, and particularly LC-MS based approaches, is the capacity for simultaneous quantification of multiple analytes. For example, detection of multiple indoleamine compounds, metabolites or conjugates or quantification of structurally or functionally related signaling molecules or plant growth regulators with a single injection. One topic which has not been widely addressed in methods for quantification of melatonin is the existence of several isomers of melatonin (Tan et al., 2012; Kocadagli et al., 2014). As a result of improved methods, several melatonin isomers (Rodriguez-Naranjo et al., 2011) have been discovered and a nomenclature proposed (Tan et al., 2012), though some controversy exists as to whether these isomers are truly melatonin isomers or instead tryptophan ethylesters (Gardana et al., 2014; Iriti and Vigentini, 2015). As research into the relevance and functions of melatonin isomers and metabolites increases, methods for the accurate, precise and sensitive quantification of this class of compounds will be instrumental in informing these studies.

## Omics

Omics technologies for studies of melatonin metabolism have exploded in the last decade. Transcriptional effects of melatonin in plants is a “hot topic” of investigation with > 150 publications mentioning transcriptomics and > 40 which used genomics or microarray approaches (Figure 9C), in addition to more targeted polymerase chain reaction (PCR)-based strategies. Diverse genes including numerous transcription factors have been described as modified by melatonin and play important roles in melatonin-mediated plant stress responses (Weeda et al., 2014). It is therefore well established that melatonin can serve as a master regulator of gene expression in plant tissues to induce protective responses in plants (Arnao and Hernández-Ruiz, 2019a). Melatonin has been found to both induce translocation of transcription factors from the cytoplasm to the nucleus, and to increase levels of transcription factors and downstream transcripts. Depending on the pathways targeted, the genes up or down regulated can include antioxidant pathways, signaling pathways, primary metabolic pathways and secondary pathways, essentially allowing for fine tuning of almost every chemical system (Weeda et al., 2014; Shi et al., 2015a; Hu et al., 2016). While transcriptomics studies have built the platform upon which further research can be built, much is still needed to fully understand the downstream effects of melatonin. The biological relevance of these gene expression changes are less well described for melatonin. It is well accepted that though transcript levels may change this does not always lead to a phenotypic effect due to the necessity for translation and post translational modifications, transport to the necessary compartment and end function of the protein and catalysis of metabolites are all required. This is increasingly being reflected in the literature as proteomic and metabolomic studies start to rise.

The question of over-emphasis on gene expression data from both targeted gene expression and untargeted transcriptomics studies is particularly highlighted in the many studies which examine the effects of a treatment or condition, e.g., metal, salt, drought on melatonin gene expression levels without quantifying the level of melatonin itself. Our understanding of melatonin biosynthetic pathways continues to improve, and with it an appreciation for the complexity and diversity built into the pathway. While this redundancy allows for significant resilience in the melatonin system, and emphasizes the importance of melatonin in plants, it makes targeted gene expression studies potentially problematic as they provide information on only the target gene. The use of transgenics has been used extensively to improve our understanding of the role of melatonin in plants with more than 50 studies published utilizing this tool. With the advent of CRISPR and more routine and specific gene editing technologies the use of transgenic overproduction or knockout lines has significant inherent value to understanding the function of the pathways in which they participate and have been instrumental in characterizing the melatonin biosynthetic pathway. Transgenic studies which are coupled with quantification of melatonin (*n* = 49) and its metabolites have been especially powerful as confirmatory tools in identifying roles for melatonin metabolites as well. This serves as an important model for consideration in the design of melatonin studies integrating genomics tools and approaches with quantification of the phenotypic effect.

To date five articles have utilized proteomics approaches to understand the mechanisms of melatonin in seed germination, chilling stress, leaf senescence, oxidative stress and fruit ripening in maize (*Zea mays* L.), crab apple (*Malus hupehensis*), tomato (*Solanum lycopersicum* L.) and Bermudagrass (*Cynodon dactylon* (L.) Pers; Wang et al., 2014; Shi et al., 2015b; Kolodziejczyk et al., 2016a,b; Sun et al., 2016). Melatonin was found to enhance polyamine metabolism, ribosome pathway, carbohydrate metabolite, photosynthesis, redox and amino acid metabolism leading to tolerance as demonstrated by improved growth and reduced cell damage and ROS accumulation in response to hydrogen peroxide treatment in Bermudagrass (Shi et al., 2015b). Melatonin-mediated changes in the proteome have been reported in response to chilling stress in maize as well as in response to melatonin application in senescing crab apple leaves, particularly with respect to stress and antioxidant related proteins (Kolodziejczyk et al., 2016b). In crab apple, modified proteins were more commonly associated with the plastid (Wang et al., 2014). Almost twice the number of metabolomics studies have been published in the area of plant melatonin research as compared to proteomics studies (*n* = 9) and have more diverse areas of interest as compared to proteomics studies. All but one have examined melatonin as a downstream metabolite mediated in the process or state studied, in contrast to proteomics and transcriptomics studies which generally focus on the impact of treatment. For example, morphogenesis in hazelnut (Erland et al., 2020b), fusarium wilt infection in watermelon (Kasote et al., 2020), low phosphorus resistance (Xu et al., 2020) and root induction in tomato (Soundararajan et al., 2017). This is a particularly exciting new area for melatonin research as it allows for the examination of the role of the melatonin naturally present in plants and has generated hypotheses of novel melatonin metabolism pathways.

## Species Studied Reflects Human Uses

**FIGURE 7:**
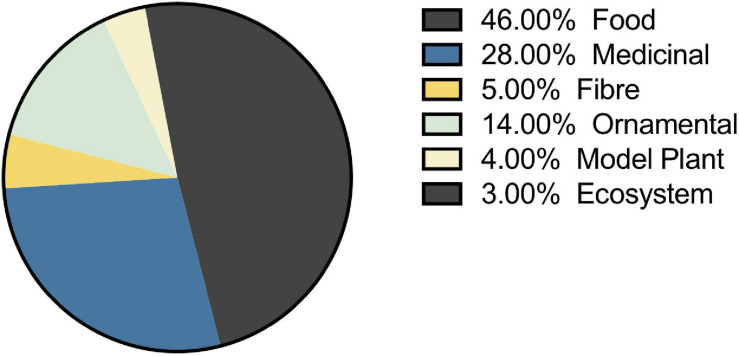
Selection of plant species for melatonin studies is largely driven by economic value. Where species can fit two categories the primary use was included for plotting.

The selection of species that have been studied in the melatonin literature has been highly influenced by economic and commercial value. Our queries of the literature identified 236 plant species representing 191 genera from 94 families for which endogenous melatonin has been quantified and its metabolism investigated (Supplementary Appendix A1 and Tables 1, 2). The greatest species diversity is represented in the Lamiaceae (*n* = 23), Leguminosae (*n* = 20), Poaceae (*n* = 18), Rosaceae (*n* = 17), and Solanaceae (*n* = 13; Table 1). Unsurprisingly, the majority of species that have been investigated are economically valuable with only 4% of studies performed on classic plant model species and 3% of studies representing plant communities or ecosystems (Figure 7). Overwhelmingly these species are of commercial importance with 46% of studies on food species and 28% of studies on medicinal plants (Figure 7). Assessment of the top 10 species investigated by number of papers finds that 7 are foods including: rice (*Oryza sativa* L.), corn (*Zea mays* L.), pear (*Pyrus communis* L.), grapes (*Vitis vinifera* L.), cucumber (*Cucumis sativa* L.), wheat (*Triticum aesativum* L.), and tomato (*Solanum lycopersicum* L.). The remaining three species include, unsurprisingly, the model species *Arabidopsis thaliana* (L. Heynh.) while the other two are medicinal species: St. John’s wort (*Hypericum perforatum* L.), which has also been considered a melatonin model species, and tobacco (*Nicotiana tabacum* L.), a valuable agricultural crop. Examination by genus shows an even greater skew toward food and feed crops with only *Arabidopsis* (*n* = 70) and *Nicotiana* (*n* = 41) remaining in the top 10 species investigated. The most well studied genera include *Solanum* (*n* = 119, nightshades), *Oryza* (*n* = 87, rice), *Malus* (*n* = 53, apples), *Brassica* (*n* = 44, mustards), *Prunus* (*n* = 41, plums, cherries, peaches, apricot, almond), *Cucumis* (*n* = 32, cucumbers and melons), *Zea* (*n* = 32, corn, maize), and *Pyrus* (*n* = 32, pears; Table 2). It is worth noting that the top ranked genus *Solanum* includes a diversity of both medicinal and food species, which is perhaps responsible for its high levels of investigation (17% of all publications). The growth and production regions of the top species investigated are also generally highly correlated; the countries from which the greatest number of publications originate, for example China and South Korea are major producers of rice, while Southern Europe including Italy and Spain are major grape and wine producing regions (Supplementary Figure 1).

**TABLE 1:** Summary of genera in which melatonin has been investigated.

Genus	Number of Papers Citing
*Solanum*	119
*Oryza*	87
*Arabidopsis*	70
*Malus*	53
*Brassica*	44
*Prunus*	41
*Nicotiana*	41
*Cucumis*	32
*Zea*	32
*Pyrus*	32
*Vitis*	29
*Triticum*	27
*Hypericum*	18
*Citrullus*	13
*Glycine*	12
*Capsicum*	12
*Manihot*	11
*Avena*	11
*Scutellaria*	10
*Citrus*	10
*Morus*	9
*Raphanus*	9
*Dracocephalum*	8
*Medicago*	8
*Lupinus*	8
*Musa*	7
*Cynodon*	7
*Fragaria*	6
*Salvia*	6
*Festuca*	6
*Thymus*	6
*Camellia*	6
*Hordeum*	6
*Helianthus*	6
*Actinidia*	6
*Ocimum*	5
*Armoracia*	5
*Gossypium*	5

*A total of 191 genera have been investigated, only genera where five or more papers have cited the genus are included.*

**TABLE 2:** Summary of families in which melatonin has been investigated.

Family	Number of Species
Lamiaceae	23
Leguminosae	20
Poaceae	18
Rosaceae	17
Solanaceae	13
Compositae	10
Brassicaceae	9
Apiaceae	5
Rubiaceae	4
Anacardiaceae	3
Myrtaceae	3
Moraceae	3
Rutaceae	3
Amaranthaceae	3
Amaryllidaceae	3
Zingiberaceae	3
Malvaceae	3
Boraginaceae	2
Actinidiaceae	2
Juglandaceae	2
Compositaceae	2
Polygonaceae	2
Araliaceae	2
Caprifoliaceae	2
Crassulaceae	2
Asparagaceae	2
Curcubitaceae	2
Betulaceae	2
Discoreaceae	2
Rhamnaceae	2
Ericaceae	2
Violaceae	2
Euphorbiaceae	2
Gentianaceae	2
Oleaceae	2

*A total of 94 families have been investigated, only families where two or more species have been investigated are displayed.*

## Major Research Themes

**FIGURE 8:**
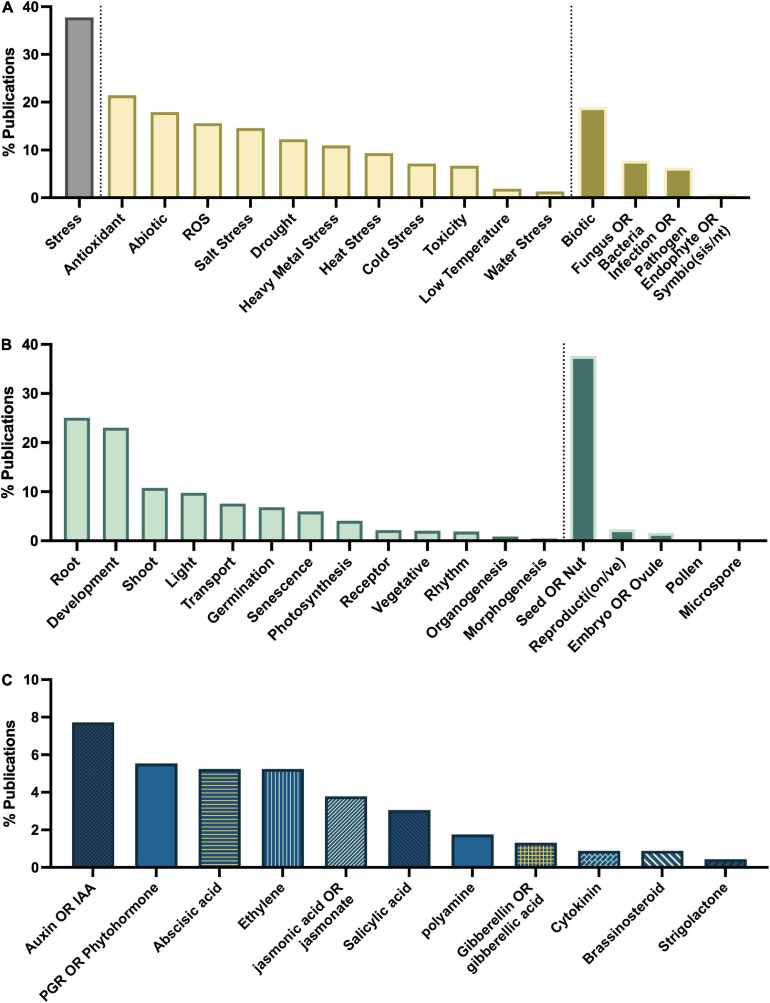
Major themes in melatonin literature number of papers on topics associated with **(A)** stress, **(B)** growth and development, **(C)** interaction with phytohormones.

**FIGURE 9:**
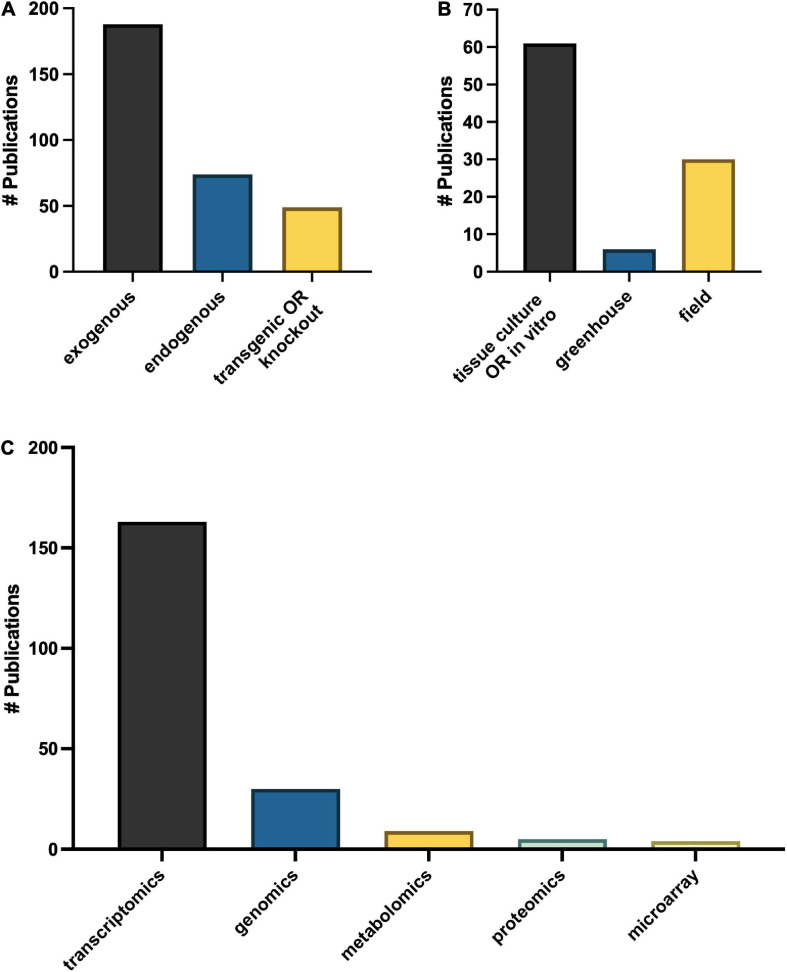
Major themes in approaches to melatonin research in plants **(A)** growth methods, **(B)** melatonin treatment type and **(C)** OMICs technologies.

**FIGURE 10:**
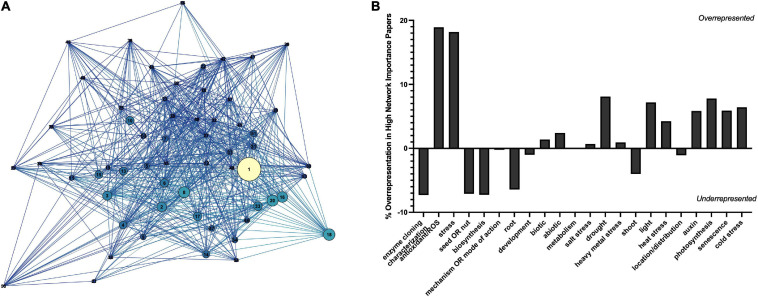
Network analysis by digital object identifier (DOI) **(A)** and representation of studies in the plant melatonin literature **(B)**. Correlation network shows relationship between top cited DOIs filtered to an in-degree cutoff of >100. Increasing node size indicates higher page rank (network importance), nodes are colored based on in-degree, the greater the number of articles citing the lighter the color. Key can be found in Supplementary Figure 3.

To determine the major themes in the literature, the dataset was queried for keywords by study type (Figure 8A), tissue type (Figure 8B), interaction with plant growth regulators (Figure 8C), environmental conditions of the study (Figure 9B), type of melatonin exposure (Figure 9A), and type of study (Figure 9C). A network analysis by digital object identifier (DOI) identified relationships between the literature themes such that some aspects are over-represented, and some are undervalued in the current publication database (Figure 10). The majority of all melatonin research focuses on exogenous application (Figure 9B). Topic area had a significant effect on the importance of a paper in the network analysis. By comparing the % representation of the query areas examined across the literature between the complete set of literature and the total literature several trends emerged (Figure 10B). Papers which investigate stress responses and particularly mention melatonin as an antioxidant were found to be overrepresented by almost 20% in the high impact papers, while papers examining morphogenetic responses such as root or shoot growth were underrepresented by journal impact. Together these analyses show that it is not the papers which make the greatest advances in the field which end up having the greatest impact on the evolution of research. Rather, specific topic areas are over amplified and driving research theme relevance. One example of the impact of publication trends is relatively few citations of the early work by Jackson (1969) or Banerjee and Margulis (1973) in modern studies that use exogenous application of melatonin to induce physiological responses and better integration of these older studies with modern data may lead to exciting discoveries. Our analysis of the literature uncovered 6 major research themes in plant melatonin metabolism.

### Major Research Theme – Abiotic Stress Responses

Queries by topic area found that the greatest areas of research interest have focused on stress responses with 37% of articles mentioning “stress” in their title or abstract, particularly with respect to melatonin’s status as an antioxidant with 21% of articles mentioning “antioxidant,” and 16% mentioning “ROS” (Figure 8A). Papers are evenly split between biotic and abiotic stress (19 and 18%, respectively; Figure 8A). The most mentioned abiotic stresses include salt (15%), drought (12% + additional 2% mentioning water stress), heavy metal (11%) and temperature stress (heat 9%, cold 7%, low temperature 2%). With more than 382 papers published on the topic of melatonin and stress this function of melatonin is one of the best described. Our meta-analysis identified 79 review papers, with several recent reviews which discuss the topic of melatonin-mediated plant stress responses (Supplementary Table 2) and we refer readers there for in-depth discussion on this topic. Melatonin has been found to have multidimensional mechanisms in mediating plant stress responses. These include induction of plant signaling cascades including MAPK (*n* = 6), calcium/calmodulin (*n* = 14), nitric oxide (*n* = 14), ROS associated signaling cascades (*n* = 3), as well as its role as a direct antioxidant (*n* = 86) and upregulation of other cellular antioxidant pathways.

The publications show that melatonin has widespread impacts on transcriptional networks inducing stress-associated transcription factors (*n* = 20) such as ZAT10/12 (*n* = 4), drought responsive element binding (DREBs; *n* = 9), CBF (*n* = 5), bZIP (*n* = 5), MYB (*n* = 7), NAC (*n* = 3) and defensive proteins, e.g., heat shock proteins (*n* = 7). Interactions with other phytohormones have also been examined (*n* = 14), particularly, abscisic acid (*n* = 19), auxin (*n* = 13), ethylene (*n* = 13), jasmonic acid (*n* = 9), polyamines (*n* = 8), salicylic acid (*n* = 6), cytokinins (*n* = 4), gibberellins (*n* = 3), and brassinosteroids (*n* = 2). As well, melatonin has been found in a significant number of studies to upregulate the ascorbate glutathione cycle, and to increase antioxidant enzymes including superoxide dismutase (SOD), catalase (CAT), and peroxidase, which have become common and non-specific screening assays in many melatonin papers with the specific enzymes mentioned in the title or abstract of 87, 72, and 75 articles, respectively and 48 mentioning “antioxidant enzyme” more generally. Likewise, quantification of ROS in plant tissues in response to melatonin treatment has also become routine, particularly with respect to H_2_O_2_ (*n* = 118), superoxide (*n* = 98), and malondialdehyde (indicator of lipid peroxidation, *n* = 85) levels which are broadly found to be reduced through melatonin treatment. Melatonin’s antioxidant activity has also been shown to mediate ROS and RNS based signaling, through quenching of H_2_O_2_ rapid signaling, one of the means by which plants perceive stresses including temperature, salinity stress and wounding (Gong et al., 2017), as well as interacting with downstream of signaling NO and ROS signaling cascades (Chen et al., 2017; Panossian et al., 2018). Melatonin has also been found to improve function and efficiency of the photosynthetic apparatus in diverse species (Szafranska et al., 2016). This mechanism has been reported to occur primarily via quenching of excess ROS species, levels of which are increased when the efficiency of the apparatus starts to break down due to stress or age leading to an inability to absorb excess light energy (Szafranska et al., 2017). Maintenance of ion homeostasis (*n* = 49) is important particularly in heavy metal and salinity stress and melatonin has been found to improve performance in several species. Melatonin-induced NO signaling (Yan et al., 2020) as well as maintenance of energy levels necessary for H + -ATPase activity and downstream effects on sodium/potassium balance have in particular been found to be modified by melatonin (Yu et al., 2018). Melatonin moves through the apoplasm and via the vasculature in plant tissues (Erland et al., 2019). The transport has been observed to occur over long distances and to occur relatively rapidly (Li H. et al., 2017). Translocation of melatonin has also been found to occur in response to temperature stress (Erland et al., 2019), which results in a diffuse pattern of melatonin localization and transport to distal organs where it functions as an antioxidant and has been found to induce cold tolerance in *Citrullus lanatus* (L.; Li H. et al., 2017) and salt tolerance in *Dracocephalum kotschyi* Boiss (Vafadar et al., 2020).

A significant body of research on melatonin in plant stress responses was generated in studies with *A. thaliana* (*n* = 37) and crop species, with the greatest number of articles reported in *Solanum* species (*n* = 55) of which 17 of those in tomato, followed by rice (*n* = 30), corn (*n* = 25), *Malus* species (*n* = 23), wheat (*n* = 21), cucumber (*n* = 16), and *Brassica* species (*n* = 15). With a growing global population, decreasing amounts of land which are traditionally suitable for agricultural cultivation, and increasing climactic uncertainty with global climate change, melatonin represents a potentially invaluable means by which to maintain or enhance agricultural production under less favorable growing conditions. The emphasis on melatonin mediated stress responses in agricultural species to date has created an interesting niche which has yet to be explored, including the potential implications of melatonin application on soil microbiomes and persistence which will be an important consideration (Madigan et al., 2019). These same factors which are having dramatic impacts on agricultural species are also impacting wild and native species around the word. These species represent culturally significant species either for ceremony, medicine or traditional food sources, as well as ecologically important species. Application of the knowledge generated in commercially or scientifically valuable species to ecologically and culturally important species holds great potential for expanding our understanding of melatonin function in plants as well as having interdisciplinary applications. The knowledge generated through these types of studies may, for example, help in determining and implementing management and conservation priorities for traditional harvest, commercial wild harvest or conservation status of species.

### Major Research Theme – Roots Are Important

A major theme in the literature is melatonin as a mediator of vegetative growth and development, most notably root system architecture (*n* = 172 articles for the term “root,” Figure 8B). In fact, the first reports of melatonin in plants, focused on melatonin in the root system reporting that melatonin treatment could control mitotic spindle development in root tips, inhibiting cellular replication and providing the first mechanism by which melatonin inhibits primary root growth (Jackson, 1969; Banerjee and Margulis, 1973). Mediation of rooting has since been reported in 76 species with the greatest number of studies having been conducted in *Arabidopsis* (*n* = 23) which have enabled mechanistic investigation of the function of melatonin in control of root growth, branching and development. Rice (*n* = 17), apple (*n* = 11), tomato (*n* = 11), wheat (*n* = 11), corn (*n* = 10), canola (*n* = 10), and pear (*n* = 10) all also have more than 10 citing papers, though these mostly report the effect of melatonin in rooting, rather than investigate the mechanisms underlying this function. In general melatonin inhibits primary root elongation, while promoting secondary or lateral root formation favoring a short more branched root network. This likely confers an advantage to these plants when nutrients are available in the higher soil layers where a more branched root system allows better nutrient acquisition.

One of the first hypothesized mechanisms of melatonin action in plants was based on the structural similarity between melatonin and auxin. This is reflected in the literature overall as ∼8% of all the original research studies queried in this meta-analysis mention the terms “auxin” or “IAA” (Figure 8C) and of the papers which mention “root” twenty-one explicitly mention auxin or IAA. The actual function, as is often the case, has turned out to be much more nuanced. The first in-depth mechanistic investigation of melatonin mediation of root system architecture in 2012 reported that melatonin stimulated lateral and adventitious root formation through an auxin independent mechanism using transgenic and reporter lines (Pelagio-Flores et al., 2012). Complimentary studies in corn reported that melatonin does not meet the threshold for auxin activity in any of the three classical auxin assays: inhibition of 1-aminocyclopropane-1-carboxylate synthase (ACC) activity, coleoptile elongation and root growth, leading the authors to reject this hypothesis (Kim et al., 2016).

In contrast several studies have found that melatonin interacts with auxin and accomplishes its actions not through mimicking auxin itself but by inducing and interacting with auxin signaling and transport mechanisms. This appears to be especially true with respect to primary root growth. Melatonin has been found to inhibit polar auxin transport through mediation of PINFORMED (PIN) auxin transport proteins, as well as auxin biosynthesis leading to reduced primary root meristem size, inhibition of primary root growth and subsequent lateral root promotion in *Arabidopsis* (Wang et al., 2016; Ren et al., 2019). In St. John’s wort (*H. perforatum*), where melatonin was first reported to have an auxin-mediated effect on root organogenesis (Murch et al., 2001), melatonin has been demonstrated to have a distinct localization pattern in primary roots compared to auxin (Erland et al., 2019) and melatonin has also been found to be induced by auxin treatment (Erland and Saxena, 2019) suggesting a further level of complication in the story. In addition to auxin, melatonin interacts with other phytohormones which are important in root growth including ethylene (*n* = 11), jasmonic acid (*n* = 10), abscisic acid (*n* = 8), polyamines (*n* = 4), salicylic acid (*n* = 3), gibberellin (*n* = 2), brassinosteroids (*n* = 2), strigolactones (*n* = 1), cytokinins (*n* = 1). Melatonin has also been found to directly induce accumulation of H_2_O_2_ leading to ROS burst and calcium-dependent induction of lateral root formation (Chen et al., 2018). The importance of mediation of H_2_O_2_ levels in root growth and morphogenesis as a function of melatonin is highlighted with H_2_O_2_ mentioned in ∼22% of all papers on the topics of “root” and “melatonin.” The importance of downstream signaling cascades has also been investigated, with calcium having been shown to play an essential role in mediating root growth (*n* = 7) including in *Mimosa pudica* L. (Ramakrishna et al., 2009). The use of transcriptomics approaches will likely continue to elucidate significant cross talk between auxin-dependent and auxin-independent mechanisms of melatonin action in plants which have been utilized extensively (*n* = 42) to study root physiology in response to melatonin treatment. Melatonin mediation of root growth can be considered to have a dual function, auxin-independent control of root branching, secondary, lateral and adventitious root growth, and auxin-dependent control of primary root growth.

Though not necessarily associated with the development of roots, captured in the “root” query is also investigation of the role of melatonin in tubers and other storage roots. Melatonin has been reported to maintain postharvest quality (*n* = 21) through its status as an antioxidant to maintain appropriate redox status of the tissues, induction of many of the same signaling cascades important in root development including calcium and MAPK signaling, as well as mediation of carbohydrate metabolism in particular (Ma et al., 2016; Hu et al., 2018). Cassava (*n* = 3) has been a particularly valuable plant systems in which these processes have been investigated.

### Major Research Theme – Light Responses

Light is perhaps the most fundamental of all environmental cues in plants, beyond driving photosynthesis it serves as a physiological and developmental cue and at high levels induces stress. Plants have both daily and seasonal responses to light and melatonin has been found to serve as a signal of the seasons in plants. As may be expected, melatonin has been well reported to help stabilize photosynthetic pigments and the photosynthetic apparatus during seasonal senescence with 41 paper published on the topic of senescence, as well as delaying leaf abscission through interaction with the eponymous phytohormone abscisic acid (Wang et al., 2012). More intriguing is the seasonal variations of melatonin in vegetative tissues such as leaves over the season without dependence on maturity stage, supporting a role for melatonin in interpretation of light signals such as photoperiod (Korkmaz et al., 2017; Zhang H. et al., 2019). The literature shows that melatonin regulates the production of photosynthetic and defensive pigments, including chlorophylls (*n* = 120), anthocyanins (*n* = 23), carotenoids (*n* = 15), and betalain (*n* = 1) helping to maintain the photosynthetic apparatus under both light stress, and conditions such as temperature stress, which reduce efficiency of the photosynthetic apparatus. In animals, melatonin plays an important role in perception of light, or rather its absence, and is colloquially referred to as the chemical expression of darkness as it mediates circadian rhythms. As a result, interest in the capacity for melatonin to mediate circadian rhythms and light perception and responses in plants has been a significant area of research interest. Melatonin was first reported to have a daily rhythm in *Chenopodium rubrum* L. in 1997 (Kolar et al., 1997) and since has been found to show rhythmic (*n* = 13) and diurnal (*n* = 11) levels in: lupine (*Lupinus albus* L.; Hernandez-Ruiz and Arnao, 2008), water hyacinth (*Eichhornia crassipes* (Mart.) Solms; Tan et al., 2007a), sweet cherry (Zhao et al., 2013), eggplant (*S. melongena*; Korkmaz et al., 2017), rice (Wei et al., 2016), tomato (Arnao and Hernández-Ruiz, 2013), St. John’s wort (Chung and Deng, 2020), and *Arabidopsis* (Li D. et al., 2020). Several melatonin metabolites have also been found to mimic melatonin expression patterns including 3-OHM (Choi and Back, 2019a) and AFMK (Tan et al., 2007a). Modulation of downstream pathways and functions associated with light dark cycles have been found to be affected by melatonin such as stomatal closure and sugar catabolism (Zhao et al., 2015). Wavelength of light is another important light signal which can be perceived by melatonin leading to downstream metabolic changes. Melatonin levels were found to have differential responses to red and blue light in *Scutellaria* species and in licorice (*Glycyrrhiza uralensis*; Afreen et al., 2006; Forsyth et al., 2020). Depending on concentration UV light can be both a signal and a stress inducer in plants, melatonin has likewise been found to have both functions, acting as an antioxidant and stress defense molecules as well as a signaling molecules. This is likely one of the reasons for which much more research has been undertaken in the UV range (*n* = 25) compared to visible light. Melatonin has previously been hypothesized to mediate plant responses through the constitutively overexpressed photomorphogenesis (COP)9 signalosome (Sanchez-Barcelo et al., 2016), an important mediator of ubiquitination and light-induced development or photomorphogenesis in plants. A recent in-depth study in *Arabidopsis* further supports this hypothesis showing that not only is the melatonin biosynthetic pathway upregulated and subsequently melatonin accumulated under UV-B exposure, but that melatonin mediated responses to UV-B exposure involves interactions with COP1 and the transcription factors HYH and HY5 (Yao et al., 2021). Though the mechanism is not well understood, the authors hypothesize that UV-B receptor 8 (UVR8), the UV-B receptor, binds to COP1 activating HY5 and HYH and their downstream gene regulation networks (Yao et al., 2021). It is unclear where in this pathway melatonin is acting, as both *Arabidopsis cop1* and *hy5* mutants show decreased responses to UV-B exposure, however, this is particularly interesting since the chromophore in the UVR8 receptor is a series of repeating tryptophan residues (Lee, 2016).

Skotomorphogenesis is the redirection of plant movement in the absence of light. The role of melatonin in skotomorphogenesis is still in its early stages of investigation and has the potential to reveal new mechanisms. In rice melatonin has been found to play a role in this process in a brassinosteroid-dependent manner (Hwang and Back, 2018). Work in rice has found that SNAT, the enzyme which converts 5HT to NAS, melatonin treatment was found to induce brassinosteroid biosynthetic genes, including the gene for the rating limiting step in brassinosteroid biosynthesis DWARF4. Knock-out of SNAT lead to skotomorphogenetic effects (i.e., the opposite of photomorphogenesis) in dark conditions, including internode shortening and increased expression of light-inducible genes, which would normally be suppressed in the absence of light (Hwang and Back, 2018).

### Major Theme – Interkingdom Communication

A unique and important feature of melatonin is its ubiquitous presence across all kingdoms of life, making melatonin an interesting candidate for mediation of interkingdom interactions. The ability for melatonin to mediate biotic stress defenses in plants has been extensively examined (*n* = 130), with melatonin being shown to improve plant resistance to fungal (*n* = 13), bacterial (*n* = 39), and viral infections (*n* = 9), through mechanisms including initial perception of threat, induction of signaling cascades and other phytohormone cascades, induction of innate immune response, and upregulation of defensive compounds. A growing area of research is the role melatonin may play in mediating symbiotic relationships between the plant root microbiome and plants (*n* = 4).

Melatonin acts as a signal to trigger downstream defense responses to pathogen challenge, particularly induction of defense-related genes and transcription factors (Lee et al., 2014a). Treatment with melatonin has been shown to be effective in increasing resistance of plants to viral, bacterial and fungal pathogens at low application levels (Moustafa-Farag et al., 2020) with the driving interest in this area being in the capacity for melatonin to improve resistance of agricultural crops with *Solanum* (*n* = 12), rice (*n* = 11), and tobacco (*n* = 7) being the most investigated species, followed by *Arabidopsis* (*n* = 6). Melatonin has been found to be able to induce defensive signaling pathways including ROS (*n* = 7) and NO signaling (*n* = 4), to upregulate phytohormones such salicylic acid (*n* = 8), jasmonic acid (*n* = 5), and ethylene (*n* = 3) and to increase production of defensive secondary metabolites such as lignans and polyphenols (*n* = 3). 2-OHM has also been reported to induce plant defenses similarly to melatonin (Lee and Back, 2019). Serotonin-phenolic conjugates are well described for their roles in the front-line mechanical defense against pathogen infection (Ishihara et al., 2008), particularly after wounding, and it has been recently hypothesized through untargeted metabolomics studies that melatonin may also form these conjugates (Erland et al., 2020a). Melatonin also upregulates transcription factors associated with plant defense and has been found to trigger plant innate immunity (Mandal et al., 2018), including pathogenesis-related (PR) proteins (*n* = 2) in response to pathogen challenge. Melatonin has also been found to induce overproduction of sugars and glycerol that are associated with plant resistance mechanisms in *Arabidopsis* (Qian et al., 2015). In some cases, pathogens have also been found to be capable of inhibition of host melatonin production, potentially enhancing melatonin susceptibility to infection, and indicating an important role for melatonin in pathogen response (Nehela and Killiny, 2018). This phenomenon has been investigated as an approach for enhancing efficiency of *Agrobacterium*-mediated transformation in recalcitrant species (Dan et al., 2015; Hou et al., 2019).

Not all interactions with prokaryotes are negative ones for plants and the function of melatonin in mediating symbioses is a new and exciting area of melatonin research. In recent years the importance of the plant microbiome has come to the forefront and has highlighted the important functions these symbioses play in improving plant health and performance, particularly under changing and stressful environmental conditions (Cordovez et al., 2019). Microbial symbionts are known to share amino acids and other nutrients with plant roots as well as to employ chemical signaling molecules, including phytohormones and NO to mediate these relationships (Whiteside et al., 2012; Cordovez et al., 2019). Melatonin is an important signaling and defense molecule in prokaryotic organisms as well as plants. Both tryptophan ethyl-ester and melatonin have been reported to be secreted by grapevine (*Vitis vinifera*, *Vitis labruscana* L.H. Bailey, and *Vitis amurensis* Rupr.) root bacterial symbionts, particularly the bacteria *Bacillus amyloliquefaciens* SB-9. In addition to secreting melatonin, *B. amyloliquefaciens* also induced melatonin biosynthesis in plant tissues through upregulation of transcripts for melatonin biosynthetic enzymes (Jiao et al., 2016). The authors also demonstrated that colonization of grapevine roots led to improved tolerance to drought and salinity stress, and reported a reduction in markers of antioxidant stress. This suggests that melatonin is an important signaling molecule mediating the symbiosis and this may be a mechanism by which bacterial symbionts improve plant stress tolerance (Jiao et al., 2016). Follow-up studies using the grape (*V. vinifera*) endosymbiont *Pseudomonas fluorescens* RG11, further demonstrated that conversion of tryptophan to melatonin using N15 labeled tryptophan in *P. fluorescens*, possibly via a tryptamine independent biosynthetic route, and demonstrated significant cross-talk between grape and bacterial melatonin biosynthetic pathways (Ma et al., 2017). Melatonin has also been found to be involved in arbuscular mycorrhizal (AM) symbioses in legumes (Zhang et al., 2020). In alfalfa, treatment with the AM *Rhizophagus irregularis* increased melatonin levels in plants, particularly upon exposure to lead stress, which was associated with increased expression of ASMT, the final enzyme in the melatonin biosynthetic pathway (Zhang et al., 2020). Increased melatonin levels led to improved plant performance through enhanced antioxidant function and reduced uptake of lead (Zhang et al., 2020). Similarly, co-application of plant growth-promoting bacteria have also found to have synergistic effects with melatonin including in faba bean (*Vicia faba* L.) and spinach (*Spinacia oleracea* L.), and have been found to improve performance under abiotic stress (Asif et al., 2020; El-Ghany and Attia, 2020). Research into a greater number of species of both plants and symbionts will help to determine how widespread these interactions are, as well as the potential signaling pathways involved. This is likely to have a significant impact on our understanding of interkingdom melatonin signaling dynamics.

The possible trafficking of melatonin between symbionts or pathogens and melatonin dates back to the first reports of melatonin in plants, where the presence of melatonin in plants was distinguished from the possibility of microbial production and contamination through the use of *in vitro* culture (Murch et al., 2000). In this way the use of *in vitro* and sterile culture has been, and will continue to be, an essential strategy in understanding and differentiating the role of melatonin in plants vs. the role of melatonin as an interkingdom signal. While more than 60 papers specify having used *in vitro* culture systems, another 28 and 7, have used field and greenhouse studies, respectively (Figure 9B). As a better understanding of the role of endosymbionts play in plant melatonin levels and functions it will be interesting to see the potential impacts of this knowledge on the existing body of literature on melatonin. This is particularly true of root symbionts which are well known for their ability to improve plant performance under stressful or low nutrient conditions, modification of root architecture, as well as improving crop traits such as yield and mass accumulation, all of which have also been associated with melatonin.

Though still an upcoming area of research, melatonin has also been found to be a signal mediating responses to wounding and insect feeding, and to mediate responses between pathogens, insect damage and plant resistance (*n* = 3). American elm (*Ulmus americana* L.) populations have been decimated by infection with dutch elm disease (*Ophiostoma ulmi*), which is a fungal pathogen whose infection is greatly facilitated by elm beetle (*Scolytus multistriatus* Marsham) feeding (Saremba et al., 2017). Resistance to the disease has been found to be dependent on appropriate balances of jasmonic acid and salicylic acid levels in the tissues as insect feeding and pathogen challenge induce antagonistic phytohormone cascades in plants. Melatonin has been proposed as one of the initial signals which signals feeding damage in plants, and which may play a role in resistance through interaction with jasmonic acid and salicylic acid signaling (Sherif et al., 2016, 2017).

### Major Research Theme – Protection of Germ Cells and Tissues

The first plant organs in which melatonin was quantified were reproductive tissues, and since these initial reports of melatonin in reproduction have continued to be by far one of the most active areas of melatonin research. More than 258 papers have investigated melatonin in seeds or nuts, and 78 in fruit, with more than half quantifying melatonin levels up to μg/g fresh weight. There is a clear role for melatonin in both the defense and direction of reproductive tissues in plants. Melatonin has also been found to have a function in pollen and microspore development (*n* = 2). Protection and defense of reproductive tissues is essential to maintain their integrity and ensure survival of populations and species. Melatonin plays an essential role in detoxifying both ROS and ammonium ions in seeds and embryos, and may also be important in controlling the progression of embryo development (*n* = 11). There appears to be a strong pattern of melatonin production with embryo development and fruit ripening, with a trade-off between melatonin and 5HT, with melatonin levels increasing during early embryo development and later stages seeing a shift toward higher 5HT content (Murch et al., 2009, 2010). In *Datura metel*, this pattern could be disrupted by cold exposure which induces a spike in melatonin levels (Murch et al., 2009). A transient spike in melatonin during seed development has also been reported in rice, which had a spike in melatonin levels associated with increased expression of melatonin biosynthetic genes in the panicle during flower development (Park et al., 2013b). In sweet cherry (*Prunus avium*) melatonin levels increased later in the season, which authors attributed to defense against high light stress and increased ROS load in the tissues (Zhao et al., 2013). Melatonin has also been found to accumulate in pollen in *Hypericum perforatum* (Murch and Saxena, 2002), and to prevent high temperature-induced pollen abortion in tomato (Qi et al., 2018).

Melatonin mediates flowering and floral timing (*n* = 18), and has been found to improve fruit and grain set, and thus yield (*n* = 62). These responses have been strongly tied to light, with melatonin potentially serving as an important signal of light quality to mediate these processes. The first plant in which this was reported is *Chenopodium rubrum* L., where exogenously applied melatonin reduced flowering when applied in the pre-during or first half of the dark phase (Kolar et al., 2003). In *Arabidopsis* the capacity for melatonin to mediate flowering has been linked to its capacity to stabilize DELLA proteins, negative regulators of gibberellins and flowering locus c, and lead to delay of flowering (Shi et al., 2016). Follow-up studies have shown that inhibition of endogenous melatonin biosynthesis by knockout of SNAT delays flowering (Lee et al., 2019), while strigolactone treatment has also been found to reduce melatonin levels leading to delay in flowering in *Arabidopsis* (Zhang Z. et al., 2019). In apple trees, melatonin levels have been found to respond to light levels in the field, particularly higher far-red and blue light levels, and that a drop in melatonin levels was associated with induction of flowers. Additionally, it was found that application of exogenous melatonin could lead to delay of flowering (Zhang H. et al., 2019). The capacity for melatonin to moderate reproductive growth has also been exploited for the improvement of postharvest quality of diverse fruit species. Melatonin has been reported to delay postharvest senescence and maintain quality of many fruits including tomato (*n* = 3), peach (*n* = 3), pear (*n* = 3), strawberry (*n* = 3), banana (*n* = 2), mango (*n* = 2), bamboo (*n* = 1), citrus (*n* = 1), kiwi (*n* = 1), and grapes (*n* = 1). This has been associated with modifications to carbohydrate and starch metabolism (*n* = 8), inhibition of ethylene and ethylene associated signals (*n* = 6) as well as melatonin’s antioxidant function (*n* = 5).

Postharvest melatonin levels have been reported to start to decrease in fruits and are possibly related to the shift in physiological function from embryo development to embryo maintenance and protection through germination. During seed germination melatonin levels have also been found to remain high and to subsequently drop as the plant matures (Korkmaz et al., 2017). Increased melatonin levels have been strongly associated with improved germination (*n* = 47) in more than 20 species, particularly under abiotic (*n* = 10) and biotic (*n* = 10) stress including salinity (*n* = 13), temperature (*n* > 10), drought (*n* = 6) and metal stress (*n* = 7). This has been associated with mechanisms including modulation of abscisic acid (*n* = 4), antioxidant function (*n* = 14) and induction of ROS signaling cascades (*n* = 4). Several in-depth transcriptomics studies have been undertaken (*n* = 8) detailing the transcriptomic cascades involved in these processes and have highlighted the roles of general pathways including energy metabolism, signal transduction, redox status and root development. Suppression of jasmonic acid biosynthesis (Hu et al., 2020) has also been found to be essential to improved germination of melon seedlings under copper stress, while melatonin was found to regulate sodium and chloride ion accumulation in rice seedlings experience salinity stress (Li X. et al., 2017). Several hydroxylated melatonin metabolites including 2-OHM (*n* = 2), 3-OHM (*n* = 2) and cyclic hydroxymelatonin (*n* = 2) are also active in improving seed germination.

### Major Research Theme – Phytohormone and Plant Signaling

Plant signaling mechanisms are less well understood than other organisms and are difficult to study due to the complexity of cell to cell communication. Melatonin is highly conserved through evolution as a fundamental signaling molecule across all forms of life. Melatonin signaling dynamics are still an evolving and exciting area of research in plants. Numerous plant signaling cascades have been hypothesized to be mediated by melatonin, including MAPK (*n* = 12), COP signalosome (*n* = 2), ROS/H_2_O_2_/NADPH oxidase signaling (*n* = 37), nitric oxide signaling (*n* = 26), Ca^2+^/CaM (*n* = 28), as well as interactions with phytohormones including auxin (*n* = 53), cytokinins (*n* = 6), salicylates (*n* = 21), jasmonates (*n* = 26), gibberellins (*n* = 9), strigolactones (*n* = 3), brassinosteroids (*n* = 6), abscisic acid (*n* = 36), polyamines (*n* = 12) and ethylene (*n* = 36). Melatonin mediates gene expression through these interconnected signaling cascades as well as mediation of microRNAs (*n* = 3) and transcription factors (*n* = 36). As with any growing field, much interest has been paid to the final effect and function of melatonin in plants; however, melatonin mediated signaling networks are the basis of melatonin function in plants and a better understanding of how plants use melatonin as a signaling molecule and how plants perceive both endogenous and applied melatonin will be fundamental in advancing the topic.

The search for plant melatonin receptors and melatonin-interacting proteins in plants is ongoing. In 2019 the first melatonin receptor CAND2-PMTR1 was described in *Arabidopsis* and reported to mediate stomatal closure in response to stress (Wei et al., 2018). The protein is a G-protein coupled receptor (GPR)50 type receptor, a group of orphan melatonin receptors and was identified based on its structural homology. The original article describes the characterization of the protein through the use of *Arabidopsis* mutant lines and binding kinetics between the protein AtCand2 and iodomelatonin (Wei et al., 2018). The authors report that loss of a functional receptor in the mutant lines leads to quenching of H_2_O_2_ production and calcium influx and propose that binding of the receptor with melatonin mediates stomatal closure (Wei et al., 2018). The protein has also been found to possess rhythmicity, which the authors associated with the previously described control of stomatal closure via ROS based signaling (Li D. et al., 2020). These results have, however, since been disputed, with efforts to repeat experiments on the receptor using transgenic, confocal microscopy (localization) and bioactivity studies being unsuccessful (Lee and Back, 2020). GPR50 proteins have previously been found to inhibit function of MT1 and MT2 receptor subtypes in animals through heterodimerization, therefore it is possible that while CAND2-PMTR1 maybe not be an authentic plant melatonin receptor, it may still be important in mediating melatonin signaling (Levoye et al., 2006). Several other melatonin interacting proteins have been reported in plants the first of which is Hyp-1, a PR 10 protein from *H. perforatum* which was shown in crystallography studies to form a complex with melatonin (Sliwiak et al., 2016). An analog has also been isolated in lupine, and was found to form a complex with both melatonin and the cytokinin zeatin (Sliwiak et al., 2018a,b). Overexpression of Hyp-1 in both tobacco (*N. tabacum*) and lettuce (*Lactuca sativa*) found that Hyp-1 localized to the nucleus, plasma membrane and cytoplasm of epidermal cells and was found to confer resistance to bacterial infection with *Agrobacterium tumefaciens* (Hou et al., 2019). Together these results suggest that Hyp-1 may be a mediator of melatonin-phytohormone cross talk to control responses to biotic challenge.

One common downstream target of initial signaling cascades is induction or repression of transcription factors. Thirty-six papers have been published which mention “transcription factor” in the title and abstract, and include numerous classes of stress-associated transcription factors including: MYB (*n* = 10), DREB (*n* = 10), bZIP (*n* = 5), ZAT (*n* = 4), WRKY (*n* = 11), bHLH (*n* = 3), NAC (*n* = 7), and HSFs (*n* = 14). Transcription factors associated with other phytohormone networks have also been reported to be induced by melatonin including the auxin response genes AUX/IAA and ethylene response factors (ERFs). Many of these transcription factors have been identified through transcriptomics studies where specific genes are generally not specified in the abstract, thus being excluded from our query. A subset have been characterized through RT-qPCR and fewer still having been validated using overexpression or knockout mutant lines (generally in *Arabidopsis*). These transcription factors have been best characterized in plant stress responses with 58% of the queried studies mentioning “stress.” The melatonin metabolite 2-OHM has also been found to be able to induce transcription factors including Myb4 and AP37 (Lee and Back, 2016b), further demonstrating important biological activities of melatonin metabolites in plants. Activation of transcription factors which then trigger downstream transcriptional activity is clearly an important function of melatonin, however, the upstream signaling mechanism which lead to induction of these transcription factors is still not known. Hopefully with the increasing interest in identification of melatonin interacting proteins this area will continue to grow.

In plants one of the first signaling cascades proposed to be induced and essential for melatonin action in plants is induction of calcium/calmodulin (Ca^2+^/CaM) signaling cascades. Melatonin is well characterized for its interaction with calcium signaling cascades in animals and is the basis for one of the first investigations of melatonin function in plants. Banerjee and Margulis (1973) reported that melatonin had “colchicine-type disruption” in onion root tips, inhibiting mitotic spindle development (Banerjee and Margulis, 1973). Colchicine is a well characterized anti-mitotic drug which inhibits calcium influx into cells leading to microtubule disruption and the authors suggest a similar mechanism for melatonin (Banerjee and Margulis, 1973). Later studies *in vitro* reported that melatonin did in fact mediate cytoskeletal rearrangements through antagonism of calmodulin using an *in vitro* mammalian system (Huerto-Delgadillo et al., 1994). A later report investigated thidiazuron-induced morphogenesis in *Echinaceae purpurea* L. suggested interaction between melatonin and calcium, as treatment with calcium transport inhibitors lead to an increase in melatonin content (Jones et al., 2007). The first direct report of dependence of melatonin action in plants was reported in 2009, where melatonin induced shoot morphogenesis in *Mimosa pudica* L. (Ramakrishna et al., 2009). These effects were found to be inhibited by treatment with the calcium channel blocker verapamil, or addition of calcium chelators to the medium (Ramakrishna et al., 2009). Since then the importance of calcium signaling continues to be demonstrated with more than 28 papers included in our query that mention calcium. While the importance of calcium in mediating plant action potentials has been established for some time (Beilby, 1984), there have been significant recent developments in the tools and transgenic lines available for the investigation of calcium signaling in the past 10 years and the importance of rapid calcium action potentials in plants continues to grow. To date several reports have described melatonin- Ca^2+/^CaM cross-talk in plant stress (*n* = 14) responses including salinity (*n* = 7), temperature (cold *n* = 3; heat *n* = 2), drought (*n* = 3) and heavy metals (*n* = 4). Given the dependence of melatonin on calcium signaling this is an area which should be a future focus.

Another signaling pathway which is highly coordinated with calcium signaling but a relatively recent development in the understanding of melatonin signaling pathways is its ability to mediate ROS signaling cascades. Melatonin has been found to induce rapid ROS signaling through interaction with the NADPH oxidase; respiratory burst oxidase homologue (RBOH), which catalyzes production of hydrogen peroxide via superoxide as an oxidation product of NADPH (Lee and Back, 2017). The importance of ROS and especially H_2_O_2_ in mediating plant responses is now well established in the literature functioning in morphogenesis, abiotic and biotic stress responses and associated physiological functions such as stomatal closure. The capacity of melatonin as an antioxidant, coupled with the growing body of literature on the important of ROS and especially H_2_O_2_ as a signaling molecule, led to interest in the interaction between the two pathways. This is an area of significant growing interest with 31 papers now published which mention “NADPH oxidase” or “RBOH” in diverse species including *Solanum* sp. (*n* = 7), particularly tomato (*S. lycopersicum*, *n* = 2), *Arabidopsis* (*n* = 4), corn (*n* = 3), rice (*n* = 3), wheat (*n* = 3), cucumber (*n* = 2), tobacco (*n* = 2), and pear (*n* = 2). ROS signals trigger diverse downstream pathways in plants, including many of the signaling cascades found to interact with melatonin including stress-associated transcription factors, MAPK, calcium signaling, and phytohormones in response to biotic and abiotic stresses (Miller et al., 2009; Gong et al., 2017). The H_2_O_2_ signals generated by RBOH have been found to occur rapidly and at the systemic scale traveling at rates of 8.4 cm/min (Miller et al., 2009). RBOH signaling exists in signaling loops which balance maintenance of sufficient levels of ROS to continue the self-propagating signal with detoxification by antioxidant systems in so called ROS signaling loops (Wrzaczek et al., 2013). Melatonin acts as an upstream signal triggering RBOH dependent H_2_O_2_ production in response to abiotic stresses including temperature, salinity and drought stress, leading to upregulation of genes associated with downstream stress-associated signaling cascades including MAPK, calcium dependent protein kinases (CDPK), heat shock proteins (HSP) and stress-associated transcription factors (Gong et al., 2017). Melatonin induction of H_2_O_2_ accumulation has also been described in the calcium-dependent control of lateral root formation in *Arabidopsis* and alfalfa (*Medicago sativa* L.; Chen et al., 2018) and seed germination in *Arabidopsis* and melon (*Cucumis melo* L.), where melatonin inhibits abscisic acid function through calcium-dependent activation of RBOH leading to H_2_O_2_ accumulation (Li H. et al., 2020). The importance of ROS and RBOH-mediated melatonin signaling has since also been established in response to biotic stress, with melatonin induction of RBOH and downstream MAPK/MKK signaling cascades in response to pathogen challenge (Lee and Back, 2017).

Mitogen-activated protein kinase serine/threonine kinases are one of the oldest signaling pathways known and are used by eukaryotic and prokaryotic organisms for the perception of environmental stimuli and control plant growth and development. They function downstream of receptor-like protein kinases (RLKs) leading to phosphorylation and induction of MAPKKK (MAPK kinase kinase), MAPKK (MAPK kinase) and finally MAPK/MPK which triggers transcription factors responsible for ultimate gene expression or secondary signal transduction pathway activation (Xu and Zhang, 2015). Melatonin-mediated responses to both biotic (*n* = 5) and abiotic (*n* = 5) cues, have been found to be dependent on MAPK signaling cascades (*n* = 11). Induction of MAPK by melatonin treatment was first reported in *Arabidopsis* and tobacco where they were found to have differential expression patterns by species. In *Arabidopsis* MPK3 and MPK6 were induced by melatonin, NAS or 2-OHM treatment. Knockout experiments identified MKK4, 5 and 7 to be the upstream MAPK kinases (MKK) mediating this response which lead to upregulation of genes associated with pathogen response and innate immunity including PRs, though induction of Hyp-1 interestingly has not been investigated (Lee and Back, 2016a). Both H_2_O_2_ and NO signaling have also been found to be activated by MAPKKK3 in *Arabidopsis* defense responses downstream of RBOH (Lee and Back, 2017), which may be triggered by melatonin-receptor binding, for example with PMTR1 (Lee and Back, 2020; Li D. et al., 2020), or potentially via interaction with the PR protein Hyp-1 though this hypothesis has yet to be investigated.

Involvement of NO signaling cascades has been reported for melatonin-induced root development (*n* = 9) and resistance to biotic (*n* = 7) and abiotic (*n* = 7) stress including bacterial (*n* = 3) and viral infection (*n* = 1), metal toxicity (*n* = 9), salt stress (*n* = 5), nutrient deficiency (*n* = 3), heat (*n* = 1), high light (*n* = 1), and maintenance of postharvest quality (*n* = 1). NO signaling in plants mediates diverse plant physiological processes, however, the structure of its generation and signaling cascades are still an area of active investigation with a common target being post-translational modification of proteins in plants (Hancock and Veal, 2020). Not only has melatonin has been found to induce NO signaling, but the physiological effects of melatonin have also been found to be dependent on induction of this signal. The downstream targets of melatonin-induced NO signals are not, however, well described. Melatonin and NO signaling interactions have been found to be associated with other signaling cascades including ROS and phytohormone signaling cascades, including ethylene (Liu et al., 2019), auxin (Wen et al., 2016), and polyamines (Ding et al., 2018), are clear. Coordination between NO and H_2_O_2_ has also been found to be important in biosynthesis of melatonin in responses to stress stimuli (*n* = 7). For example, in rice both NO and H_2_O_2_ are required for activity of melatonin biosynthetic enzymes, and apparently also for trafficking of the intermediates between cellular compartments in response to cadmium exposure (Lee et al., 2017).

Melatonin is now well accepted as a master regulator and mediator of many other phytohormone networks. It plays a central role to balance, fine-tune and direct more classical phytohormone signaling cascades with the particular action and response varying depending on the function (Erland et al., 2015; Arnao and Hernández-Ruiz, 2018a, 2020). Melatonin may drive the balance between well-known phytohormone pairs including auxins and cytokinins in morphogenesis (Murch et al., 2001) and jasmonic acid and salicylic acid in responses to pathogen and insect attack (Sherif et al., 2016). Melatonin interaction with auxin in particular, and in interaction with ethylene (*n* = 11), polyamines (*n* = 4), jasmonic (*n* = 9) and abscisic acid (*n* = 8), as well as interactions with Ca^2+^/CaM (*n* = 11), H_2_O_2_ (*n* = 37), and NO signaling (*n* = 9) have been found to be essential to melatonin-mediated effects on root growth. Interactions between zeatin and melatonin, potentially at the Hyp-1 protein may be important in pathogen defense (Sliwiak et al., 2016), while interaction with gibberellin (*n* = 2) and abscisic acid (*n* = 4) mediates seed germination.

An interesting novel signaling mechanism for melatonin is its interaction with lipid signaling. Melatonin has been found to mediate fatty acid composition in various species, which has been primarily investigated with respect to membrane integrity in response to abiotic stress (*n* = 15). Melatonin has recently been reported to be a regulator of endoplasmic reticulum (ER) stress (Ozgur et al., 2016) and to mediate phospholipid levels (Yu et al., 2018). Phospholipids are synthesized and trafficked between the chloroplast and ER where they can induce signaling cascades which have previously been described to be induced by treatment with the melatonin precursor 5HT, mimicking red light exposure (Chandok and Sopory, 1994). Melatonin has been reported to increase both phosphatidic acid (PA) and phosphatidylinositol (PI) levels in *Ipomea batatas* (L.; Yu et al., 2018), and several reports have found that melatonin levels are increased in response to red light exposure (Forsyth et al., 2020) suggesting that perhaps a similar mechanism could occur for melatonin. This will certainly be an exciting novel area of plant melatonin research.

## Discussion

### Bias in the Literature

A significant, but to date, unacknowledged bias exists within plant melatonin literature. To investigate our anecdotal observations of bias we undertook a network analysis of all of the original research papers meeting our search terms, and which possess a DOI to identify the papers which are the most influential in the field of melatonin literature as a function of their citation network (Figure 10A). An examination of the papers with highest network impact (as defined by degree > 100, i.e., min 100 papers cited or citing; Figure 10) was then undertaken. Our results show poor agreement between our qualitative keystone papers and the papers with highest network importance, suggesting that the papers with the greatest influence on the literature are not those which have made keystone discoveries in the field. As the network was created using DOI as the identifier for papers, papers which do not have a DOI were excluded from the analysis and this is responsible for the absence of several of these keystone papers including Hattori et al. (1995), which was one of two papers first quantifying melatonin in foods, Murch et al. (1997), the first report of melatonin quantification in vegetative plant materials and Jackson (1969), the first report of melatonin activity in the plant system. To further investigate what factors are driving high network importance we examined the journal title and topics in which these papers were published. The keystone papers show a generally well distributed list of publication titles, while the high network importance papers are heavily skewed to publication in the Journal of Pineal Research (JPR) with 76% of High Network Importance but only 33% of the Keystone Papers, while an even lower proportions of all papers, only 20%, are published in JPR (Supplementary Figure 2). Interestingly, journals such as the Journal of Experimental Botany (JXB) which represent the next greatest proportion of high impact papers 9% is not included in the keystone papers and represents only a fraction of all papers. Examination of the author network reveals several author collaborator research hubs.

## Conclusion

Plant melatonin research is a rapidly expanding field of research and will continue to be an active area which will have applications for ecosystem function, agriculture and human health. We have identified several areas of bias within the literature, and highlighted key gaps. In particular, future studies should investigate non-medicinal and non-crop species as well as emphasizing not just the function of melatonin, but also the mechanisms of melatonin action, as well as characterization of melatonin signaling cascades, melatonin receptors and melatonin interacting proteins represent new opportunities in plant melatonin research.

## Author Contributions

LE and SM participated in design, analysis, presentation, and writing of manuscript. Both authors contributed to the article and approved the submitted version.

## Conflict of Interest

The authors declare that the research was conducted in the absence of any commercial or financial relationships that could be construed as a potential conflict of interest.

## Supplementary Material

Supplementary Appendix Species in which melatonin has been investigated, listed in order by family. The top 10 most investigated species are shaded. A total of 236 species and 94 families have been investigated.

Supplementary Data 1 Complete bibliography with original research articles included in this study and query terms.

Supplementary Data 2 Complete bibliography of review articles included in this study and query terms.

Supplementary Data 3 Complete list of species queries in original research articles included in this study.

Supplementary Figure 1 Country of corresponding author for original research articles, where country is specified filtering for countries with > 5 publications. Numbers in legend indicate number of publications.

Supplementary Figure 2 Top journals publishing plant melatonin research by **(a)** highest network importance, **(b)** Keystone papers included in Figure 1, and **(c)** all original research articles.

Supplementary Figure 3 Top papers included in DOI network (in degree cut-off of 100) with numbers in table corresponding to node numbering also displayed in Figure 10.

Supplementary File 1 Summary of methods used for literature review.
