# Supplementary material for: A Systematic Review of Melatonin in Plants: An Example of Evolution of Literature
Source: Front Plant Sci. 2021 Jun 18;12:683047. doi: 10.3389/fpls.2021.683047 (PMC8270005; doi:10.3389/fpls.2021.683047)
Supplement: Supplementary Appendix — Species in which melatonin has been investigated, listed in order by family. The top 10 most investigated species are shaded. A total of 236 species and 94 families have been investigated. [file Data_Sheet_2.docx]

Appendix 1. Species in which melatonin has been investigated, listed in order by family, top 10 most investigated species are shaded. A total of 236 species and 94 families have been investigated.

| **Family** | **Genus** | **Species** | **Authority** | **Scientific Name in Melatonin Literature if other than accepted name** | **Common Name** | **# Papers citing** |
| --- | --- | --- | --- | --- | --- | --- |
| Actinidiaceae | *Actinidia* | *deliciosa* | (A.Chev.) C.F.Liang & A.R.Ferguson |  | kiwi | 1 |
| Actinidiaceae | *Actinidia* | *chinensis* | Planch. |  | golden kiwi | 1 |
| Amaranthaceae | *Chenopodium* | *rubrum* | L. |  | red goosefoot | 3 |
| Amaranthaceae | *Spinacia* | *oleracea* | L. |  | spinah | 1 |
| Amaranthaceae | *Amaranthus* | *tricolor* | L. |  | amaranth | 1 |
| Amaryllidaceae | *Allium* | *cepa* | L. |  | onion | 2 |
| Amaryllidaceae | *Scadoxus* | *multiflorus* | (Martyn) Raf. | *Haemanthus katherinae* | African blood lily | 1 |
| Amaryllidaceae | *Leucojum* | *aestivum* | L. |  | summer snowflake | 1 |
| Anacardiaceae | *Mangifera* |  |  |  | mango | 2 |
| Anacardiaceae | *Pistacia* | *lentiscus* | L. |  | Mastic tree | 1 |
| Anacardiaceae | *Pistacia* | *palaestina* | Boiss. |  | terebinth, turpentine tree | 1 |
| Apiaceae | *Pimpinella* | *anisum* | L. |  | (green) anise | 2 |
| Apiaceae | *Angelica* | *sinensis* | (Oliv.) Diels |  | dong quai | 1 |
| Apiaceae | *Coriandrum* | *sativum* | L. |  | coriander | 1 |
| Apiaceae | *Foeniculum* | *vulgare* | Mill. |  | fennel | 1 |
| Apiaceae | *Apium* | *graveolens* | L. |  | celery | 1 |
| Apocynaceae | *Catharanthus* |  |  |  | periwinkle | 4 |
| Araliaceae | *Panax* | *ginseng* | C.A.Mey. |  | ginseng | 1 |
| Araliaceae | *Panax* | *notoginseng* | (Burkill) F.H.Chen | *panax notoginsneg* | Chinese ginseng | 1 |
| Arecaceae | *Phoenix* | *dactylifera* | L. |  | date palm | 2 |
| Asparagaceae | *Asparagus* | *aphyllus* | L. |  | Mediterranean asparagus | 1 |
| Asparagaceae | *Ruscus* | *aculeatus* | L. |  | Butcher's broom | 1 |
| Azioaceae | *Carpobrotus* | *edulis* | (L.) N.E.Br. |  | ice plant | 1 |
| Berberidaceae | *Epimedium* | *brevicornu* | Maxim. |  | barrenwort | 1 |
| Betoideae | *Beta* | *vulgaris* | L. |  | beet | 3 |
| Betulaceae | *Betula* | *platyphylla* | Sukaczev |  | which birch | 1 |
| Betulaceae | *Corylus* |  |  |  | hazelnut, filbert | 1 |
| Bigoniaceae | *Catalpa* | *bungei* | C.A.Mey |  | Manchurian catalpa | 1 |
| Boraginaceae | *Arnebia* | *euchroma* | (Royle) I.M.Johnst. |  | pink arnebia | 1 |
| Boraginaceae | *Phacelia* | *tanacetifolia* | Benth. |  |  | 1 |
| Brassicaceae | *Arabidopsis* | *thaliana* | L. Heynh. |  | mouse ear cress | 70 |
| Brassicaceae | *Brassica* | *napus* | L. |  | canola, rapeseed | 16 |
| Brassicaceae | *Raphanus* |  |  |  | radish | 9 |
| Brassicaceae | *Brassica* | *oleracea* | L. |  | cabbage, kohlrabi | 6 |
| Brassicaceae | *Armoracia* | *rusticana* | P.Gaertn., B.Mey. & Scherb. |  | horseradish | 5 |
| Brassicaceae | *Brassica* | *nigra* | (L.)K.Koch |  | black mustard | 2 |
| Brassicaceae | *Brassica* | *juncea* | (L.) Czern. |  |  | 2 |
| Brassicaceae | *Sinapis* | *alba* | L. | *Brassica hirta* | white mustard | 1 |
| Brassicaceae | *Lepidium* | *sativum* | L. |  | garden cress | 1 |
| Bromeliaceae | *Ananas* | *comosus* | (L.) Merr. |  | pineapple | 1 |
| Caprifoliaceae | *Lonicera* | *japonica* | Thunb. |  | Japanese honeysuckle | 1 |
| Caprifoliaceae | *Lonicera* | *etrusca* | Santi |  | Etruscan honeysuckle | 1 |
| Caryophyllaceae | *Stellaria* | *aquatica* | (L.) Scop. | *Malachium aquaticum* | giant chickweed | 1 |
| Circubitaceae | *Citrullus* | *lanatus* | (Thunb.) Matsum. & Nakai | *Citrullus lanatus* | watermelon | 13 |
| Cistaceae | *Cistus* | *albidus* | L. |  | grey-leaved cistus | 1 |
| Compositaceae | *Helianthus* | *annuus* | L. |  | sunflower | 6 |
| Compositaceae | *Echinacea* | *purpurea* | (L.) Moench |  | echinacea | 1 |
| Compositae | *Tanacetum* | *parthenium* | (L.) Sch.Bip. |  | feverfew | 3 |
| Compositae | *Carthamus* | *tinctorius* | L. |  | safflower | 3 |
| Compositae | *Stevia* | *rebaudiana* | (Bertoni) Bertoni |  | candyleaf | 2 |
| Compositae | *Silybum* | *marianum* | (L.) Gaertn. |  | milk thistle | 2 |
| Compositae | *Dendranthema* | *morifolium* | (Ramat.) Tzevelev |  | chrysanthemum | 1 |
| Compositae | *Matricaria* | *chamomilla* | L. | *Matricaria chamomila* | chamomile | 1 |
| Compositae | *Galinsoga* | *parviflora* | Cav. |  | potato weed | 1 |
| Compositae | *Achillea* | *millefolium* | L. |  | yarrow | 1 |
| Compositae | *Tripleurospermum* | *disciforme* | (C.A.Mey.) Sch.Bip. |  | mayweed | 1 |
| Compositae | *Saussurea* | *orgaadayi* | Khanm. & Krasnob. |  |  | 1 |
| Convolulaceae | *Ipomoea* | *batatas* | (L.) Lam. |  | sweet potato | 2 |
| Convolvulaceae | *Evolvulus* | *alsinoides* | (L.)L. |  | slender dwarf morning glory | 1 |
| Cornaceae | *Cornus* | *officinalis* | Siebold & Zucc. | *Coruns officinalis* | Japanese cornel dogwood | 1 |
| Crassulaceae | *Sedum* | *roseum* | (L.) Scop. | *Rhodiola rosea* |  | 1 |
| Crassulaceae | *Rhodiola* | *crenulata* | (Hook.f. & Thomson) H.Ohba |  |  | 1 |
| Cucurbitaceae | *Momordica* | *charantia* | L. |  | bitter melon | 1 |
| Cupressaceae | *Chamaecyparis* | *obtusa* | (Siebold & Zucc.) Endl. |  | false cyprus | 1 |
| Curcubitaceae | *Cucumis* | *sativa* | L. |  | cucumber | 28 |
| Curcubitaceae | *Cucumis* | *melo* | L. |  | melon | 4 |
| Discoreaceae | *Dioscorea* | *alata* | L. |  | purple yam | 1 |
| Discoreaceae | *Dioscorea* | *cayennensis* | Lam. |  | yellow yam | 1 |
| Ephedraceae | *Ephedra* | *foeminea* | Forssk. | *ephedra campylopoda* |  | 1 |
| Equisetaceae | *Equisetum* | *arvense* | L. |  | horsetail | 1 |
| Ericaceae | *Pyrola* | *decorata* | Andres | *Pirola decorata* | luxxiancao | 1 |
| Ericaceae | *Vaccinium* | *corymbosum* | L. |  | high bush blueberry | 1 |
| Euphorbiaceae | *Manihot* | *esculenta* | Crantz |  | cassava | 11 |
| Euphorbiaceae | *Hevea* | *brasiliensis* | (Willd. Ex. A.Juss.) Müll.Arg |  | Rubber Tree | 1 |
| Gentianaceae | *Gentiana* | *macrophylla* | Pall. |  | qin jiao, large leaf gentian | 1 |
| Gentianaceae | *Gentiana* | *scabra* | Bunge |  | gentian | 1 |
| Hypericaceae | *Hypericum* | *perforatum* | L. |  | St. John's wort | 18 |
| Juglandaceae | *Carya* | *cathayensis* | Sarg. |  | hickory | 2 |
| Juglandaceae | *Juglans* | *regia* | L. |  |  | 1 |
| Lamiaceae | *Scutellaria* | *baicalensis* | Georgi |  |  | 2 |
| Lamiaceae | *Scutellaria* | *racemosa* | Pers. |  |  | 1 |
| Lamiaceae | *Scutellaria* | *lateriflora* | L. |  |  | 1 |
| Lamiaceae | *Ocimum* | *basilicum* | L. |  | basilicum | 5 |
| Lamiaceae | *Dracocephalum* |  |  |  |  | 4 |
| Lamiaceae | *Rosmarinus* | *officinalis* | L. |  | rosemary | 3 |
| Lamiaceae | *Dracocephalum* | *moldavica* | L. |  | moldavian dragonhead | 2 |
| Lamiaceae | *Dracocephalum* | *kotschyi* | Boiss. |  |  | 2 |
| Lamiaceae | *Prunella* | *vulgaris* | L. |  | self-heal/heal all | 2 |
| Lamiaceae | *Melissa* | *officianlis* | L. |  | lemonbalm | 2 |
| Lamiaceae | *Thymus* | *vulgaris* | L. |  |  | 2 |
| Lamiaceae | *Leonurus* | *japonicus* | Houtt. |  | motherwort | 1 |
| Lamiaceae | *Mentha* | *pulegium* | L. |  | pennyroyal | 1 |
| Lamiaceae | *Scutellaria* | *amoena* | C.H.Wright |  | huang-qin | 1 |
| Lamiaceae | *Salvia* | *nemorosa* | L. |  | woodland sage | 1 |
| Lamiaceae | *Salvia* | *reuteriana* | Boiss. | *Salvia reuterana* |  | 1 |
| Lamiaceae | *Perilla* | *frustecens* | (L.) Britton |  | shiso | 1 |
| Lamiaceae | *Artemisia* | *tridentata* | Nutt. |  |  | 1 |
| Lamiaceae | *Origanum* | *majorana* | L. |  | marjoram | 1 |
| Lamiaceae | *Thymus* | *fedtschenkoi* | Ronniger |  |  | 1 |
| Lamiaceae | *Thymus* | *daenensis* | Celak. |  |  | 1 |
| Lamiaceae | *Cannabis* | *sativa* | L. |  | marijuana | 1 |
| Lamiaceae | *Salvia* | *miltiorrhiza* | Bunge |  | red sage, danshen | 1 |
| Lauraceae | *Laurus* | *nobilis* | L. |  | bay laurel | 1 |
| Leguminosae | *Glycine* | *max* | (L.) Merr. |  | soybean | 12 |
| Leguminosae | *Lupinus* |  |  |  | lupin | 8 |
| Leguminosae | *Medicago* | *sativa* | L. |  | alfalfa | 8 |
| Leguminosae | *Trigonella* | *foenum-graecum* | L. |  | fenugreek | 3 |
| Leguminosae | *Glycyrrhiza* | *uralensis* | Fisch. |  | chinese licorice | 3 |
| Leguminosae | *Pisum* | *sativum* | L. |  | pea | 2 |
| Leguminosae | *Lens* | *culinaris* | Medik. |  | lentil | 2 |
| Leguminosae | *Cajanus* | *cajan* | (L.) Millsp. |  | pigeon pea | 2 |
| Leguminosae | *Arachis* | *hypogaea* | L. |  | peanut | 2 |
| Leguminosae | *Sophora* | *flavescens* | Aiton |  | shrubby sophora | 1 |
| Leguminosae | *Desmodium* | *styracifolium* | (Osbeck) Merr. |  |  | 1 |
| Leguminosae | *Pueraria* | *montana var lobata* | (willd.) Sanjappa & Pradeep | *Pueraria lobata* | kudzu | 1 |
| Leguminosae | *Senna* | *alexandrina* | Mill. | *Cassia angustifolia* | senna | 1 |
| Leguminosae | *Senna* | *tora* | (L.) Roxb. |  | tora | 1 |
| Leguminosae | *Sesbania* | *grandiflora* | (L.) Pers. | *Sesbania glandiflora* | vegetable hummingbird | 1 |
| Leguminosae | *Sesbania* | *sesban* | (L.) Merr. |  | sesban | 1 |
| Leguminosae | *Vigna* | *radiata* | (L.) R.Wilczek |  | mung bean | 1 |
| Leguminosae | *Pongamia* | *pinnata* | (L.) Pierre |  | Indian beech, Pongam oiltree | 1 |
| Leguminosae | *Vigna* | *unguiculata* | (L.) Walp. |  | cowpea | 1 |
| Leguminosae | *Vicia* | *faba* | L. |  | faba bean, broad bean | 1 |
| Linaceae | *Linum* | *usitatissimum* | L. |  | flax | 3 |
| Loranthaceae | *Taxillus* | *chinensis* | (DC.) Danser |  | mulberry mistletoe | 1 |
| Lytraceae | *Punica* | *granatum* | L. |  | pomegranite | 1 |
| Malvaceae | *Gossypium* | *hirsutum* | L. |  | cotton | 5 |
| Malvaceae | *Tilia* | *platyphyllos* | Scop. |  | large leaved linden | 1 |
| Malvaceae | *Malva* | *parviflora* | L. |  | little mallow | 1 |
| Meliaceae | *Melia* | *azedarach* | L. | *Melia axedarach* | Chinaberry | 1 |
| Mniaceae | *Plagiomnium* | *cuspidatum* | (Hedw.) T.J. Kop. |  | woodsy thyme moss | 1 |
| Monimiaceae | *Peumus* | *boldus* | Molina |  | boldo | 1 |
| Moraceae | *Morus* | *alba* | L. | *mori albae* | white mulberry, sang ye | 2 |
| Moraceae | *Ficus* | *carica* | L. |  | fig | 1 |
| Moraceae | *Morus* | *nigra* | L. |  | black mulberry | 1 |
| Moringaceae | *Moringa* | *oleifera* | Lam. |  | drumstick tree | 1 |
| Musaceae | *Musa* |  |  |  | banana | 7 |
| Myrtaceae | *Acca* | *sellowiana* | (O.Berg) Burret | *Feijoa sellowiana* | pineapple guava | 1 |
| Myrtaceae | *Myrtus* | *communis* | L. |  | myrtle | 1 |
| Myrtaceae | *Syzygium* | *samaragense* | (Blume) Merr. & L.M.Perry |  | java apple | 1 |
| Oleaceae | *Olea* | *europae* | L. | *Olea europaea* | olive | 2 |
| Oleaceae | *Phillyrea* | *latifolia* | L. |  | green olive, mock privet | 1 |
| Orchidaceae | *Dendrobium* | *sonia* |  |  |  | 1 |
| Paeoniaceae | *Paeonia* | *lactiflora* | Pall. |  | peony | 2 |
| Papaveraceae | *Papaver* | *somniferum* | L. |  | poppy | 1 |
| Passifloraceae | *Passiflora* | *edulis* | Sims | *Passiflora incarnata* | passionfruit | 1 |
| Pedaliaceae | *Sesamum* | *indicum* | L. |  | black sesame | 1 |
| Pentaphylacaceae | *Adinandra* | *nitidia* | Merr. Ex H.L.Li | *Babreum coscluea* | shiya tea | 1 |
| Phyllanthaceae | *Baccaurea* | *ramiflora* | Lour. |  | Burmese grape | 1 |
| Piperaceae | *Piper* | *nigrum* | L. |  | black pepper | 1 |
| Plumbaginaceae | *Limonium* | *bicolor* | (Bunge) Kuntze |  | sea lavender | 2 |
| Poaceae | *Avena* | *sativa* | L. |  | oat | 11 |
| Poaceae | *Cynodon* | *dactylon* | (L.) Pers. |  | bermudagrass | 7 |
| Poaceae | *Zea* | *mays* | L. |  | corn, maize | 32 |
| Poaceae | *Triticum* | *aesativum* | L. |  | wheat | 27 |
| Poaceae | *Hordeum* | *vulgare* | L. |  | barley | 6 |
| Poaceae | *Festuca* |  |  |  | fescue | 3 |
| Poaceae | *Panicum* | *virgatum* | L. |  | switchgrass | 3 |
| Poaceae | *Lolium* | *perenne* | L. |  | perennial ryegrass | 2 |
| Poaceae | *Agrostis* | *stolonifera* | L. |  | bentgrass | 2 |
| Poaceae | *Festuca* | *arundinacea* | Schreb. |  | tall fescue | 2 |
| Poaceae | *Carex* | *duriuscula subsp rigescens* | C.A.Mey. | *Carex rigescens* |  | 2 |
| Poaceae | *Phalaris* | *canariensis* | L. |  | canary grass | 1 |
| Poaceae | *Elymus* | *nutans* | Griseb. |  |  | 1 |
| Poaceae | *Festuca* | *ovina* | L. |  | hard fescue | 1 |
| Poaceae | *Zoysia* | *japonica* | Steud. |  | zoysiagrass | 1 |
| Poaceae | *Paspalum* | *notatum* | Flüggé |  | bahiagrass | 1 |
| Poaceae | *Leymus* | *chinensis* | (Trin.) Tzvelev |  | wheatgrass | 1 |
| Poaceae | *Oryza* | *sativa* | L. |  | rice | 87 |
| Polygalaceae | *Polygala* | *tenuifolia* | Willd. |  | yuan zhi | 1 |
| Polygonaceae | *Rheum* | *palmatum* | L. |  | ornamental rhubarb | 1 |
| Polygonaceae | *Reyoutria* | *multiflora* | (Thunb.) Moldenke | *Caulis polygonammultiflorum* |  | 1 |
| Polygonaeceae | *Fagopyrum* | *tataricum* | (L.) Gaertn. |  | buckwheat | 1 |
| Pontederiaceae | *Eichhornia* | *crassipes* | (Mart.) Solms |  | water hyacinth OR eichhornia crassipes | 1 |
| Ranunculaceae | *Coptis* | *chinensis* | Franch. |  | goldthread | 1 |
| Resedaceae | *Ochradenus* | *baccatus* | Delile |  | taily weed | 1 |
| Rhamnaceae | *Ziziphus* | *jujuba* | Mill. |  | jujube red date | 1 |
| Rhamnaceae | *Ziziphus* | *spina-christi* | (L.) Desf. |  | Christ's thorn | 1 |
| Rosaceae | *Pyrus* | *communis* | L. |  | pear | 32 |
| Rosaceae | *Malus* | *domestica* | Borkh. |  | apple | 7 |
| Rosaceae | *Malus* | *hupehensis* | (Pamp.) Rehder |  | tea crabapple, chinese crabapple | 6 |
| Rosaceae | *Fragaria* |  |  |  | strawberry | 6 |
| Rosaceae | *Prunus* | *avium* | (L.) L. |  | sweet cherry | 5 |
| Rosaceae | *Prunus* | *persica* | (L.) Batsch |  | peach | 4 |
| Rosaceae | *Malus* | *zumi* | (Matsum.) Rehder |  | crabapple | 4 |
| Rosaceae | *Prunus* | *cerasus* | L. |  | tart/sour/dwarf cherry | 3 |
| Rosaceae | *Prunus* | *domestica* | L. |  | plum | 3 |
| Rosaceae | *Eriobotrya* | *japonica* | (Thunb.) Lindl. |  | loquat | 2 |
| Rosaceae | *Prunus* | *canescens* | Bois |  | grey-leaf cherry | 1 |
| Rosaceae | *Prunus* | *mahaleb* | L. |  | Mahaleb or St. Lucie cherry | 1 |
| Rosaceae | *Prunus* | *armeniaca* | L. |  | apricot | 1 |
| Rosaceae | *Prunus* | *dulcis* | (Mill.) D.A.Webb | *Prunus amydalus* | almond | 1 |
| Rosaceae | *Crataegus* | *rhipidophylla* | Gand. | *Crataegus oxyacantha* | English hawthorne | 1 |
| Rosaceae | *Crataegus* | *azarolus* | L. | *Crataegus aronia* |  | 1 |
| Rosaceae | *Rubus* | *sanctus* | Schreb. |  | holy bramble | 1 |
| Rubacieae | *Coffea* | *canephora* | Pierre ex A.Froener |  | coffee | 3 |
| Rubiaceae | *Rubia* | *tenuifolia* | d'Urv |  | narrow leaved madder | 1 |
| Rubiaceae | *Gardenia* |  |  |  | gardenia | 1 |
| Rubiaceae | *Ophiorrhiza* | *pumila* | Champ. ex Bent. |  |  | 1 |
| Rubiaceae | *Uncaria* | *rhyncophylla* | (Miq.) Miq. Ex Havil. |  | cat's claw herb | 1 |
| Rutaceae | *Citrus* | *aurantium* | L. |  | bitter orange | 2 |
| Rutaceae | *Phellodendron* | *amurense* | Rupr. |  | Amur cork tree | 1 |
| Rutaceae | *Citrus* | *medica* | L. | *Citrus medico* | citrus | 1 |
| Saliceae | *Populus* |  |  |  | poplar | 3 |
| Santalaceae | *Osyris* | *alba* | L. |  | osyris | 1 |
| Sapindaceae | *Litchi* | *chinensis* | Sonn. |  | litchi | 2 |
| Sapotaceae | *Chrysophyllum* | *cainito* | L. |  | star apple | 1 |
| Smilacaceae | *Smilax* | *aspera* | L. |  | sarsaparille | 1 |
| Solanaceae | *Solanum* | *lycopersicum* | L. | *Lycopersicon esculentum* | tomato | 27 |
| Solanaceae | *Nicotiana* | *tabacum* | L. |  | tobacco | 24 |
| Solanaceae | *Capsicum* | *annuum* | L. |  | pepper | 12 |
| Solanaceae | *Solanum* | *tuberosum* | L. |  | potato | 4 |
| Solanaceae | *Nicotiana* | *benthamiana* | Domin |  |  | 3 |
| Solanaceae | *Lycium* | *barbarum* | L. |  | Chinese wolfberry OR goji | 2 |
| Solanaceae | *Solanum* | *pimpinellifolium* | L. | *Lycopersicon pimpinellifolium* | currant tomato | 2 |
| Solanaceae | *Solanum* | *melongena* | L. |  | eggplant | 1 |
| Solanaceae | *Solanum* | *americanum* | Mill. | *Solanum nigrum* | black nightshade | 1 |
| Solanaceae | *Solanum* | *elaeagnifolium* | Cav. | *Solanum alaeagnifolium* | silverleaf nightshade | 1 |
| Solanaceae | *Datura* | *metel* | L. |  | datura | 1 |
| Solanaceae | *Withania* | *somnifera* | (L.) Dunal |  | ashwagandha | 2 |
| Solanaceae | *Cyphomandra* | *betacea* | (Cav.) Sendtn. |  | tamarillo | 1 |
| Theaceae | *Camellia* | *sinensis* | (L.) Kuntze |  |  | 6 |
| Ulmaceae | *Ulmus* | *americana* |  |  | elm | 2 |
| Umbelliferaceae | *Ligusticum* | *porteri* | J.M.Coult & Rose |  | osha | 1 |
| Urticaceae | *Pilea* |  |  |  |  | 1 |
| Valerianaceae | *Valeriana* | *officinalis* | L. |  | valerian | 2 |
| Verbenaceae | *Lantana* | *camara* | L. |  | lantana | 1 |
| Violaceae | *Viola* | *philippica* | Cav | *Viola philipica* | arrow-leaf violet | 1 |
| Violaceae | *Viola* | *odorata* | L. |  | sweet violet | 1 |
| Vitaceae | *Vitis* | *amurensis* | Rupr. |  | Amur grape | 1 |
| Vitaceae | *Vitis* | *labruscana* | L.H. Bailey |  | wild grape | 1 |
| Vitaceae | *Vitis* | *vinifera* | L. |  | grape | 29 |
| Zingiberaceae | *Zingiber* | *officinale* | Roscoe |  | ginger | 2 |
| Zingiberaceae | *Curcuma* | *aeruginosa* | Roxb. |  |  | 1 |
| Zingiberaceae | *Elettaria* | *cardamomum* | (L.) Maton | *Eletaria cardamomum* | green cardamum | 1 |
| Zygophyllaceae | *Fagonia* | *indica* | Burm.f. |  | Indian fagonia | 2 |
