# Supplementary figures and images for: A Systematic Review of Melatonin in Plants: An Example of Evolution of Literature
Source: Front Plant Sci. 2021 Jun 18;12:683047. doi: 10.3389/fpls.2021.683047 (PMC8270005; doi:10.3389/fpls.2021.683047)

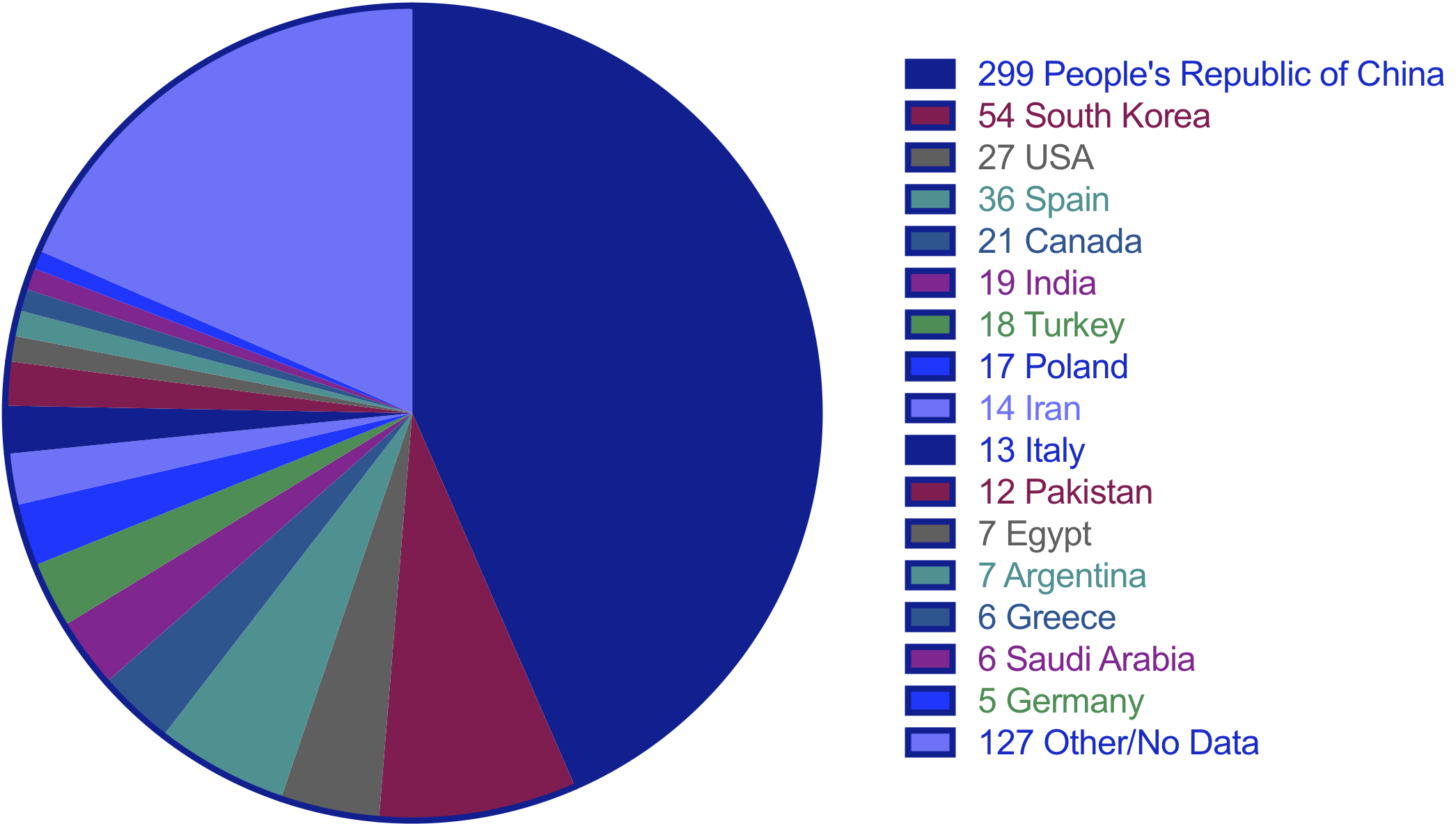

Supplement: Supplementary Figure 1 — Country of corresponding author for original research articles, where country is specified filtering for countries with > 5 publications. Numbers in legend indicate number of publications. [file Image_1.TIFF]

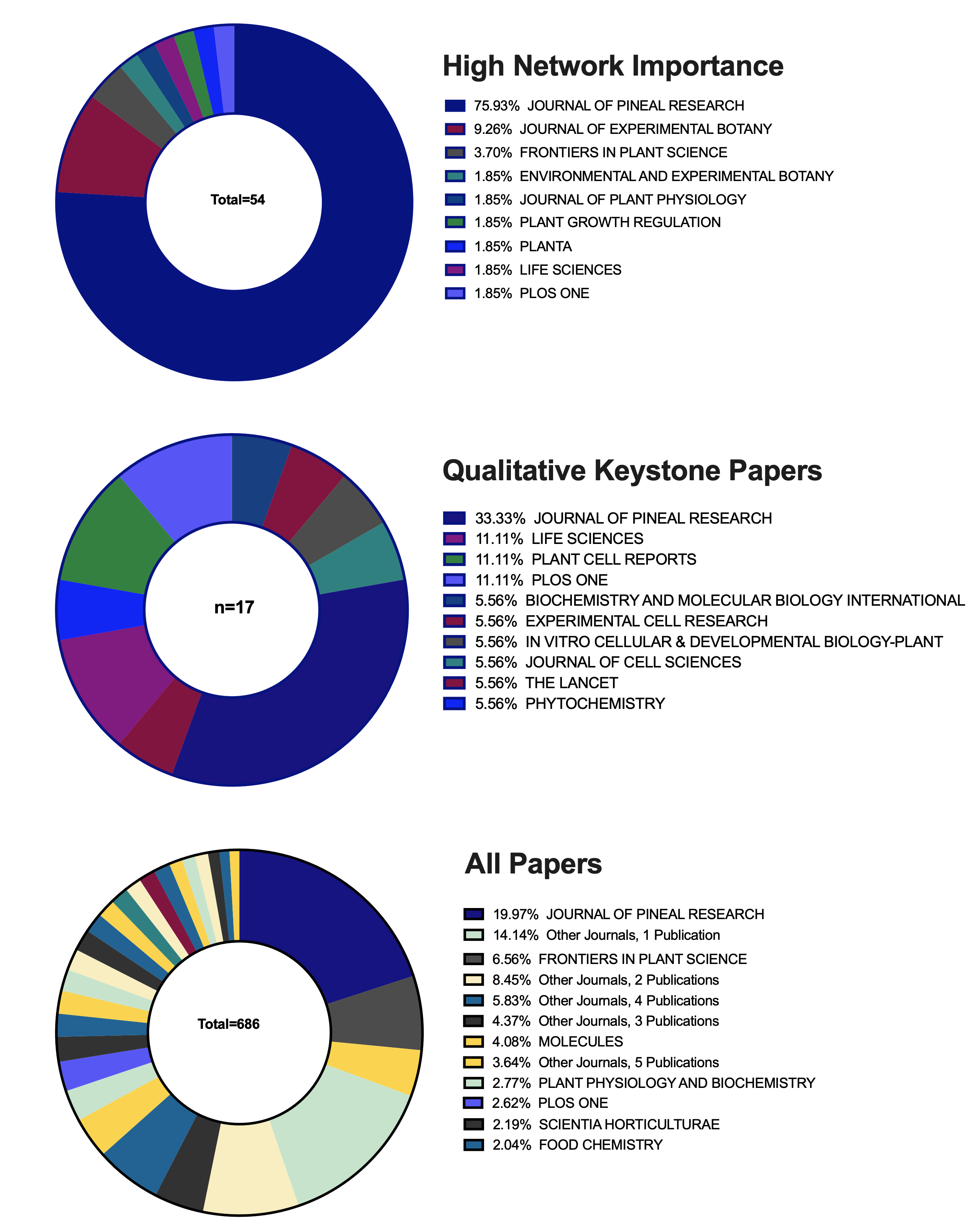

Supplement: Supplementary Figure 2 — Top journals publishing plant melatonin research by (a) highest network importance, (b) Keystone papers included in Figure 1, and (c) all original research articles. [file Image_2.TIFF]

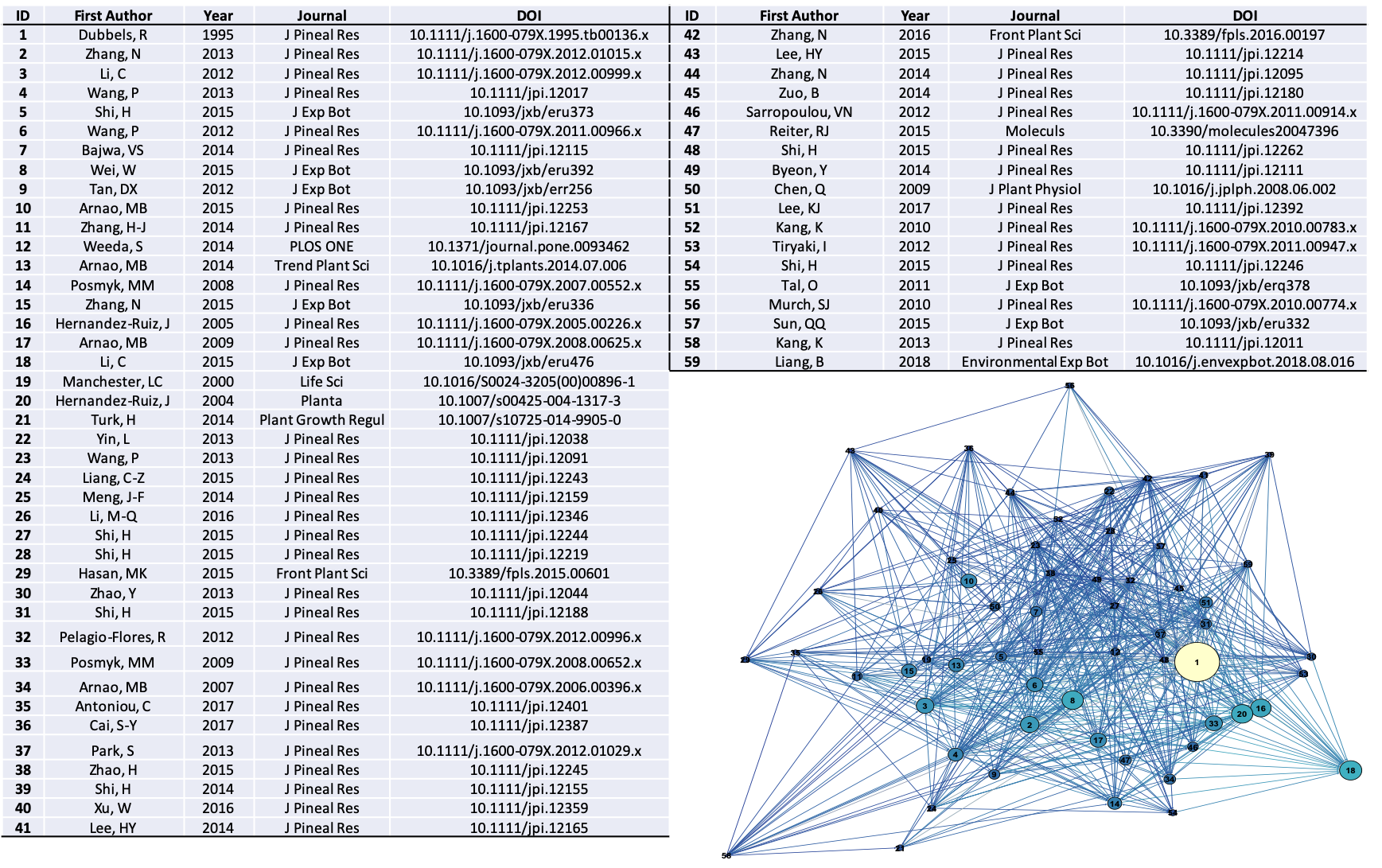

Supplement: Supplementary Figure 3 — Top papers included in DOI network (in degree cut-off of 100) with numbers in table corresponding to node numbering also displayed in Figure 10. [file Image_3.TIFF]
