# Supplementary material for: A Systematic Review of Melatonin in Plants: An Example of Evolution of Literature
Source: Front Plant Sci. 2021 Jun 18;12:683047. doi: 10.3389/fpls.2021.683047 (PMC8270005; doi:10.3389/fpls.2021.683047)
Supplement: Supplementary File 1 — Summary of methods used for literature review. [file Data_Sheet_1.PDF]

Methods of Literature Review

Protocol and Registration

A systematic review was conducted in accordance to the Preferred Reporting Items for Systematic Reviews and Meta-Analyses (PRISMA) guidelines(Moher et al., 2009) and is registered in the Open Source Framework (osf.io/ng8x3) Figure 4 outlines the PRISMA review process employed in this study.

Information Sources and Search

Web of Science™ (WoS, Clarivate Analytics) was used as the primary information source and was accessed on January 4-7, 2021. Records were initially identified by searching the terms TOPIC: "melatonin" AND "plants" with a PUBLICATION DATE range of 1993-2021, including only the CITATION INDEXES: "Science Citation Index Expanded" AND "Emerging Sources Citation Index". Articles were then further sorted by DOCUMENT TYPE: Article OR Review. Papers known by the authors which were not captured in the original search, but deemed relevant based on the eligibility criteria outlined in 2.3 were then manually added. This included papers from the relatively new melatonin-focused journal Melatonin Research which are not indexed in Web of Science, relevant papers published prior to the 1993 date cut-off (n=2) and one paper published in the journal The Lancet in 1997.

Screening and Eligibility

Records were sorted into to two spreadsheets in Excel™ (Microsoft): original research or reviews, hereafter referred to as only original research and reviews and assessed for eligibility manually. Articles were retained only if they were available in English and the primary focus was melatonin in the plant system e.g. review papers which mention melatonin as one of a list of treatments contributing to stress tolerance or papers which looked at treatment of animals with plants in which melatonin was quantified were not included. After screening and eligibility 694 original research articles and 99 review articles were retained for further investigation. Full records including metadata, citation records and citing research were exported for the eligible articles or manually curated in the case of those records not acquired from WoS as detailed above.

Data collection, items and query terms

Data items were collected in two manners (1) directly from the record metadata (Journal Title, Publication Data, Author List, Cited Papers, digital object identifier (DOI), First Author, Last Author) and (2) through queries of the title and abstract. The former was performed using the standard meta-data retrieved from the initial search.

Titles and abstracts of all records were queried and classified as 1 – contains the term, or 0 – does not contain the term, using functions in Excel™. Queries included species investigated, study type ("greenhouse", "in vitro", "field"), melatonin type ("exogenous", "endogenous", "transgenic" OR "knockout"), topic area, analytical methods ("LC" OR "liquid chromatography" OR "liquid-chromatography", "GC" OR "gas chromatography" OR "gas-chromatography", "MS" OR "mass spectrometry" OR "mass-spectrometry", "UV" OR "PDA" or "DAD", "fluorescence" OR "FLD", "electrochemical" OR "ECD", "ELISA" OR "enzyme linked immunosorbent assay" OR "EIA" OR "enzyme immunoassay", "RIA" OR

“radioimmunoassay”), and -OMICS approaches (“proteomics” OR “proteome”, “genomics” OR “genome”, “transcriptomics” OR “transcriptome” OR “RNA-Seq”, “microarray”, “metabolomics” OR “metabolome”). Supplementary Table 1 contains the complete list of query terms for original research articles, Supplementary Table 2 the complete list for review articles, and Supplementary Table 3 contains the full list of species queries.

Risk of Bias

To mitigate the risk of author bias the full datasets generated in this search are included as Supplementary Tables 1 and 2. To examine potential biases in the existing literature, papers of greatest importance to the field were assessed in two ways (1) Qualitative assessment of the literature by the authors to determine a list of keystone papers in the history of plant melatonin research termed “Qualitative Keystone Papers” and visualized in Piktochart (www.piktochart.com) and (2) A network analysis of papers as described in the following section, which included only publications with an associated DOI.

Network Analysis

To identify papers which are the most influential in the literature a citation network was generated as follows. All records in the original research dataset were identified by DOI along will all cited articles and compiled into an adjacency list which was uploaded in GEPHI (v.0.9.2, www.gephi.org) open graph visualization software. A directed graph network was generated with network visualization using force atlas 2 (Bastian et al., 2009; Jacomy et al., 2014). Degree range was then filtered to 100-279. Page rank was calculated using default settings and selected for ranking of overall network importance of the filtered DOIs (n = 59) in the network as it accounts for not just raw number of citations but the importance of the citing papers (e.g. number of citations each of the citing papers has; Brin and Page, 1998). This dataset was termed “High Network Importance”. Metadata was then extracted for this dataset and queried in the same manner as the overall datasets.

To allow for comparison of author networks between the whole body of literature and the papers identified as being of highest network impact two author citation networks were created: one for the full original research dataset and the second just for the high impact papers dataset. Both included the first author and last author of all papers in the dataset and the first author of papers cited. Only first author was chosen as this is the output format of cited references in WoS. Networks were visualized as for the DOI network using force atlas 2, and filtered to include only authors with in-degree range >100.

Synthesis of Query Results

Query results were either summed and percent of papers including the search term calculated based on total number of records in the dataset (n=694 for original research, n=99 for reviews) or used for sorting to further examine records which contained specified search terms by metadata (e.g. number of publications utilizing a particular analytical technique per year) using the pivot tables function in Excel. Data were visualized in Prism (v9.0, GraphPad Software LLC).

Method References

Bastian, M., Heymann, S., and Jacomy, M. (2009). Gephi: an open source software for exploring and manipulating networks. *International AAAI Conference on Weblogs and Social Media*.

Brin, S., and Page, L. (1998). The anatomy of a large-scale hypertextual Web search engine. *Comput Networks Isdn* 30, 107–117. doi:10.1016/s0169-7552(98)00110-x.

Jacomy, M., Venturini, T., Heymann, S., and Bastian, M. (2014). ForceAtlas2, a continuous graph layout algorithm for handy network visualization designed for the Gephi software. *PLoS ONE* 9, e98679. doi:10.1371/journal.pone.0098679.
